# MicroRNA expression in serum samples of sulfur mustard veterans as a diagnostic gateway to improve care

**DOI:** 10.1371/journal.pone.0194530

**Published:** 2018-03-22

**Authors:** Sedigheh Gharbi, Shahriar Khateri, Mohammad Reza Soroush, Mehdi Shamsara, Parisa Naeli, Ali Najafi, Eberhard Korsching, Seyed Javad Mowla

**Affiliations:** 1 Department of Biology, Faculty of Sciences, Shahid Bahonar University of Kerman, Kerman, Iran; 2 Janbazan Medical and Engineering Research Center (JMERC), Tehran, Iran; 3 National Institute of Genetic Engineering and Biotechnology, Tehran, Iran; 4 Department of Molecular Genetics, Faculty of Biological Sciences, Tarbiat Modares University, Tehran, Iran; 5 Molecular Biology Research Center, Baqiyatallah University of Medical Sciences, Tehran, Iran; 6 Institute of Bioinformatics, University Hospital of Münster, University of Münster, Münster, Germany; ENEA Centro Ricerche Casaccia, ITALY

## Abstract

Sulfur mustard is a vesicant chemical warfare agent, which has been used during Iraq-Iran-war. Many veterans and civilians still suffer from long-term complications of sulfur mustard exposure, especially in their lung. Although the lung lesions of these patients are similar to Chronic Obstructive Pulmonary Disease (COPD), there are some differences due to different etiology and clinical care. Less is known on the molecular mechanism of sulfur mustard patients and specific treatment options. microRNAs are master regulators of many biological pathways and proofed to be stable surrogate markers in body fluids. Based on that microRNA expression for serum samples of sulfur mustard patients were examined, to establish specific microRNA patterns as a basis for diagnostic use and insight into affected molecular pathways. Patients were categorized based on their long-term complications into three groups and microRNA serum levels were measured. The differentially regulated microRNAs and their corresponding gene targets were identified. Cell cycle arrest, ageing and TGF-beta signaling pathways showed up to be the most deregulated pathways. The candidate microRNA miR-143-3p could be validated on all individual patients. In a ROC analysis miR-143-3p turned out to be a suitable diagnostic biomarker in the mild and severe categories of patients. Further microRNAs which might own a link to the biology of the sulfur mustard patients are miR-365a-3p, miR-200a-3p, miR-663a. miR-148a-3p, which showed up only in a validation study, might be linked to the airway complications of the sulfur mustard patients. All the other candidate microRNAs do not directly link to COPD phenotype or lung complications. In summary the microRNA screening study characterizes several molecular differences in-between the clinical categories of the sulfur mustard exposure groups and established some useful microRNA biomarkers. qPCR raw data is available via the Gene Expression Omnibus https://www.ncbi.nlm.nih.gov/geo/query/acc.cgi?acc=GSE110797.

## Introduction

Sulfur mustard [bis (2-chloroethyl) sulfide] is a vesicant chemical warfare agent, which has been used during world war I (1914–1918) and in recent times during 8-year Iraq-Iran-war (1980–1988) [1]. The sulfur mustard victims are severely affected throughout their whole life if they survived this injury. Especially in Iran it is a humanitarian challenge to cure and help theses many forgotten people in society.

The sulfur mustard exposure of the human body causes a systemic disease. We will introduce for the whole phenotype in the following sections the acronym 'SMV'. As an alkylating agent, sulfur mustard reacts with a lot of macromolecules in the human body. Especially DNA and RNA in the cells are the obvious targets of sulfur mustard and this interaction is responsible for many clinical manifestations of sulfur mustard exposure [2, 3]. The guanine base of nucleotides is the preferential site of sulfur mustard action which can lead to mono- and bifunctional adducts [2]. These adducts are preferentially found at the N7 position of guanine but rarely at O6 position. Although very uncommon, O6-(2-ethylthioethyl guanine) is the most critical DNA lesion as human DNA repair system is unable to remove it [2]. Bifuntional sulfur mustard adducts including intra- or inter strand crosslinks occurred in nearly 17% of the sulfur mustard alkylations resulting in single or double strand DNA breaks [2]. In vitro and in vivo experiments showed that, beyond a certain amount of DNA alkylation, the direct consequences are apoptosis or necrosis [4].

In a comprehensive study, Khateri and colleagues [1] showed that of the approximately 100,000 military and civilian Iranian people, who had been exposed to sulfur mustard, a large group of 34,000 people suffer up to now from its chronic effects, mainly located in their lung, eyes and skin. The lung lesions turned up to be the most frequently diagnosed complication of SMV (42.5%) [1]. Indeed, the mucosal membrane of the respiratory tract provides an enormous surface area for sulfur mustard action. Additionally, the moistness nature of the lung surface makes it an ideal site of sulfur mustard reactivity [1] as body temperature and moisture enhance the destructive effects of sulfur mustard [5].

Pathological studies, pulmonary function test (PFT) and other clinical examinations showed that COPD (Chronic Obstructive Pulmonary Disease) is the main long-term lung disorders among sulfur mustard patients. But the COPD phenotype observed in SMV patients is different from other conventional COPDs resulting from causes such as smoking. Serological findings regarding SMV patients showed that, among all proposed mechanisms for COPD, oxidative stress and apoptosis are more probable to be involved in COPD pathogenesis [6]. Because of the complicated nature of the lung disorder in SMV, the exact biological mechanism of this disease is still an active field of researches [6]. In this regard, screening studies are necessary to define the pattern of molecular drivers.

qPCR arrays are a reliable and sensitive option in investigating the expression pattern of a large amount of microRNAs in the peripheral blood system as surrogate markers for systemic changes in the lung and further organs [7]. The post-transcriptional regulatory role of microRNAs directly influences many important biological pathways. These non-coding RNAs are stable enough to be detected in biological samples such as serum, a prerequisite for becoming a clinical application [7, 8]. They also show constant and invariable expression patterns under storage conditions, which makes them more appropriate over other biomarkers [8]. Their stability against RNase activity, which is a big problem in transcriptional studies, is an additional advantage [8]. The pathophysiological deregulation of microRNA markers has already been described in a similar disease phenotype of the pulmonary disorder COPD and emphysema [9–11]. Additionally, it has been described that serum has sufficiently significant microRNA signatures being useful to distinguish different disease states [7, 12].

Because of the phenotypic similarity of lung lesions of SMV with COPD cases, the GOLD procedure (Global Initiative for Chronic Obstructive Lung Disease) is adopted to evaluate the severity of pulmonary lesions in these patients on a clinical level [1] and to categorize the patient cohort.

The objectives of this study were to find out if the microRNA expression is differentially expressed in serum samples of SMV compared to normal ones, and which biological pathways are linked to the differentially expressed microRNAs. This can be exploited in defining therapeutic strategies for the clinical procedures or diagnostic applications. Here, microRNAs might offer an appropriate and reliable diagnostic alternative, which can be a substitute for more inaccurate diagnostic methods of SMV like high resolution CT scan and PFT. Finally, this study should help to reconfirm some preliminary aspects of previous research efforts.

## Materials and methods

### Patients

**Fig 1:**
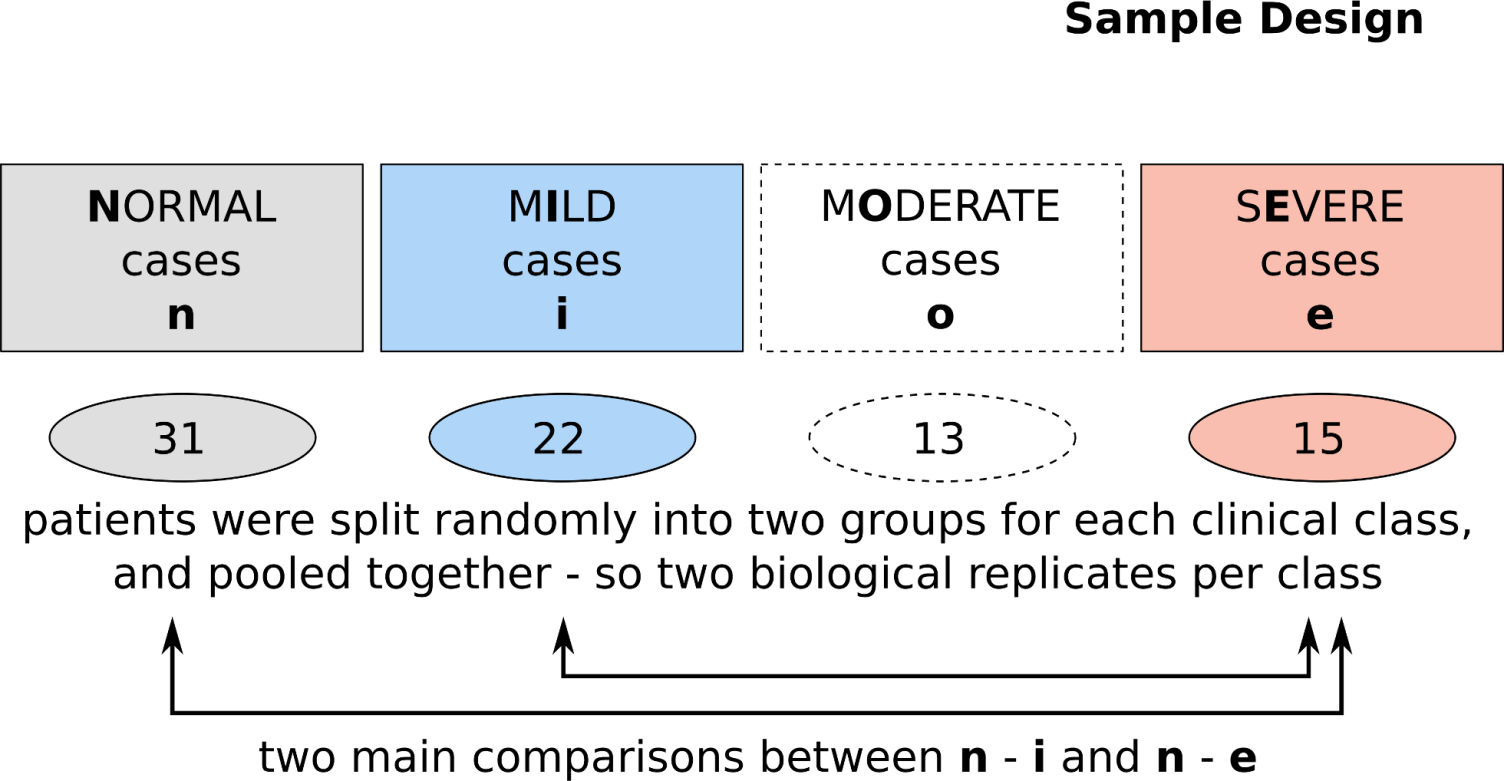
Patient cohort. Participants were categorized into the four classes of normal, mild, moderate and severe based on the history of exposure to sulfur mustard, PFT and other clinical examinations. The samples of the screening study were randomly pooled into two biological replicates and measured.

Eighty-four male participants, including SMV patients and age- and gender-matched healthy persons, were enrolled in this case and control study in 2011 / 2012. An overview of the screening procedure can be seen in Fig 1. Due to the variability of the symptoms in the SMV phenotype, all the patients were chosen from a homogeneous group of veterans who had been exposed to high doses of mustard gas during a gas attack in February 1986. The participants in the control group were volunteers, who had not been exposed to any chemical substances during their lifetime either in war zone or their work place. The exclusion criteria for both groups were based on smoking, chronic disease of lung, skin and eyes and also malignancies. The average age of patients in this study was 47.5 ± 3.6 years ranging from 40 to 64 years.

The grouping into normal study participants and mild, moderate and severe SMV patients was done according to the severity of their eye, lung and skin lesions. Spirometry test values of the lung (forced expiratory volume—FEV1, forced vital capacity—FVC) were used to categorize patients based on their lung function. The classification of the eye and skin functionality was done according to Khateri et al. [1]. The study was approved by the research ethics committee of Janbazan Medical and Engineering Research Center (JMERC 89. E.B.101). Written informed consents were obtained from all participants.

### Peripheral blood sampling

Eight milliliters of peripheral blood were collected in BD Vacutainer tubes with clot activator and gel (BD, Plymouth, UK). The time point of sampling was always early in the morning. The samples were placed at room temperature for 20 minutes and serum was separated by centrifugation at room temperature [13]. Each serum sample was stored at -80°C until further analysis.

### RNA extraction and quantitative PCR

The patients from the mild, moderate and severe groups were randomly pooled into two, disjunct biological replicates. Equal volumes of sera were mixed together and the pooled samples were subjected to RNA extraction. Total RNA was extracted from 250 microliters of each pooled serum samples using miRNeasy mini kit (Qiagen, Germany) based on manufacture`s protocol. To increase the yield of microRNA extraction, MS2 RNA (Roche Applied Science, Germany) was added to each sample before adding QIAzole. 1.5 microliters of each RNA samples were reverse transcribed in a volume of 10 microliters. This amount of RNA input has the least inhibitory effect based on our previous experiments on serum samples assessing the inhibitory effect of RNA input on microRNA expression [14]. cDNA synthesis was performed in a two-step protocol of RNA polyadenylation followed by universal cDNA synthesis (Exiqon, Denmark). Thereafter, the quantitative PCR panel was run using SYBR green master mix (Exiqon, Denmark) by the Light Cycler 480 (Roche, Germany). The expression pattern of 752 microRNAs was assessed for each sample using the Exiqon made panels (microRNA Ready-to-Use PCR, Human panel I+II, V2.R).

### Data processing and analysis

**Fig 2:**
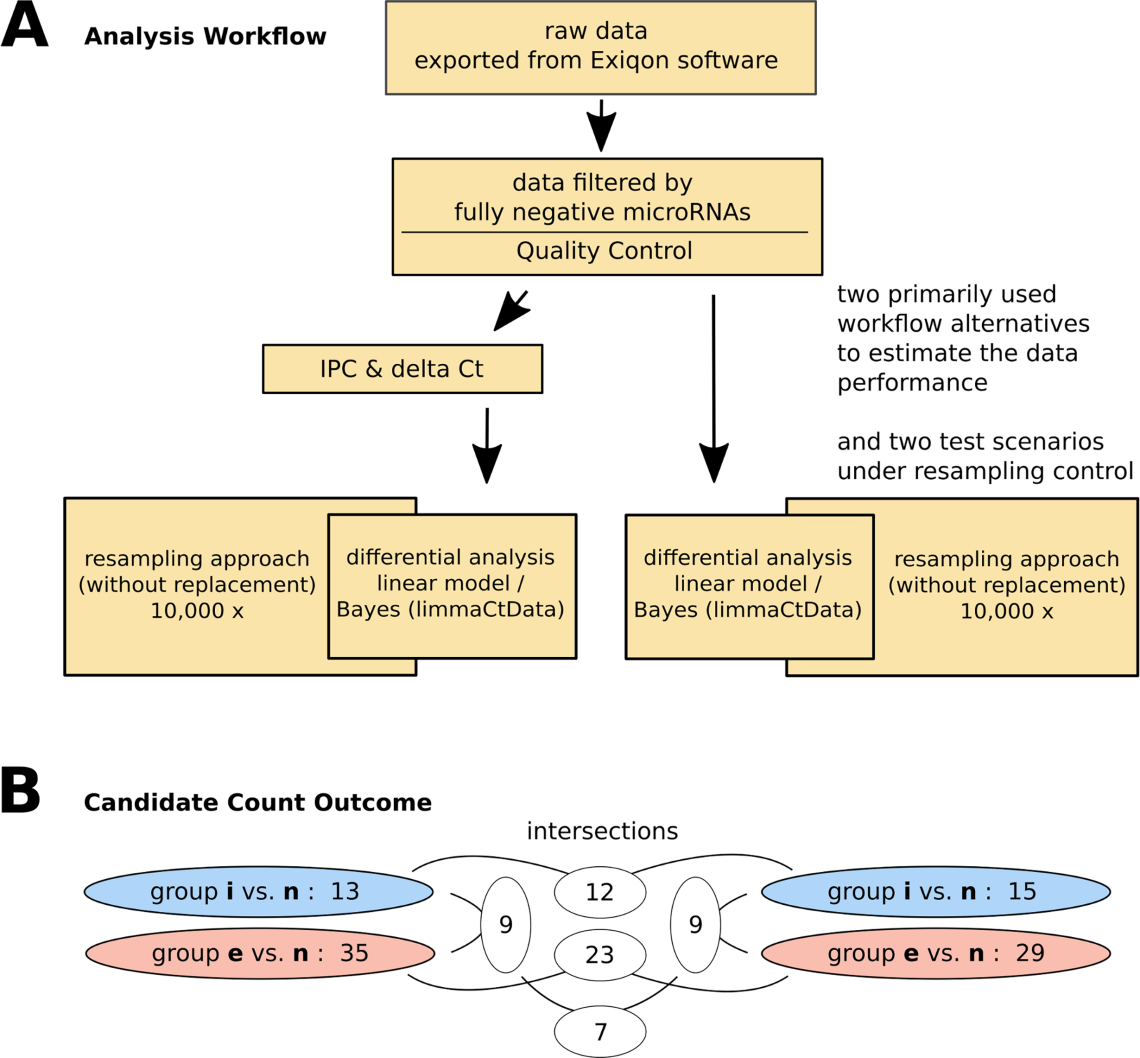
Comparative differential microRNA analysis workflow including sampling controls. The panel (**A**) shows that two different analysis approaches were applied. On the left side the IPC and delta Ct method followed by linear model / Bayes based differential analysis was performed while on the right side a pure linear model / Bayes approach was used based on the results of the pilot analysis. The relevance of both approaches was tested by a resampling approach. The right branch was finally chosen and basis for the discussion. In panel (**B**) the results of all the alternative tests on either the normal-mild or the normal-severe comparisons are shown. The results on the right side indicate that in the normal-mild comparison 15 and in the normal-severe comparison 29 microRNAs are stable on a 5% significance level after applying the resampling control. The numbers on white background indicate the remaining candidates after the intersections were performed. The result denotes a good consistency between left and right procedure.

Totally, due to the complexity of disease and to increase the power of the analyses, three different strategies based on the R (3.2.3) Bioconductor (3.2) packages of limma (3.26.9) [15], HTqPCR (1.24.0) [16] and R CRAN package of MCMC (0.9–5) [17] were tested to get insight into the performance of the methods. Among these, HTqPCR package was the most reliable package, and had the advantage of several test statistics and normalization procedures and was therefore finally chosen for the presented analyses (Fig 2A).

The quality control and the differential analysis was done with the HTqPCR package functions. Based on the quality control (i.a. S1 and S2 Figs) a raw and IPC/delta Ct analysis was performed. The differential analysis was therefore also performed in two different approaches. In one branch the raw data was directly used for a linear model-based Bayes approach (HTqPCR: limmaCtData) followed by a resampling control (see section 'Sampling procedure'). In parallel, and as an outer control to the HTqPCR differential analysis workflow, a simple normalization approach, well established in the qPCR community as well as recommended by Exiqon company, was applied on the expression sets (see next paragraph). The significant differences were again evaluated on an alpha error of 5 percent applying a linear model-based Bayes approach. Like in the first procedure, a resampling (permutation) analysis was added on the selected data workflow to establish a sampling p value denoting the reliability of the results. An overview on the used HTqPCR functions and the code of the custom functions is given in S1 Functions.

### Data normalization by delta Ct method

The approach is a well-established method [18–20]. To keep the Exiqon specific peculiarities a proposed workflow of the company was used. Briefly, after raw data collection, inter-plate calibration and internal control normalization were performed. The used predefined inter-plate calibrators, annotated as UniSp3 IPC (inter plate calibrator), should have the same expression value across all panels. in which a standard deviation of less than 0.05 is necessary to pass this quality control step. For each panel, a calibration factor was calculated based on the Exiqon's manual.

Additionally, six different small RNAs including three small nucleolar RNAs and three microRNAs exist in each panel including: SNORD38B (ENSG00000207421), SNORD49A (ENSG00000277370), U6 snRNA (ENSG00000206625), and miR-103a-3p (MIMAT0000101), miR-423-5p (MIMAT0004748) and miR-191-5p (MIMAT0000440). These factors are provided as reference genes for the delta Ct normalization method [6].

In a separate study, we evaluated the stability of these six recommended internal controls and found miR-103a-3p, miR-423-5p and miR-191-5p (named r103, r191, r423 in the raw data) as the most consistent and reliable internal controls among others [21]. So the mean value of these three stable microRNAs was finally used as reference gene in calculating the delta Ct value.

### Sampling procedure

A sampling method was applied on the delta Ct respectively the raw data to show the reliability of obtained results. Specifically, a resampling procedure without replacement was applied (permutation, per group, on all microRNAs of a group). This algorithm is known to be a very reliable and test distribution conformable procedure to control the alpha error [22–24]. A permutation number of 10,000 times was chosen. The p value was calculated by the fraction of permutation approaches better than the original p value without resampling.

### Pathway enrichment analysis

The differential microRNAs were mapped to the corresponding gene targets to reveal the associated pathways. miRTarbase [25] is one of several tools that is using validated microRNA-target interactions instead of predicted microRNA-gene interactions resulting in a higher relevance in vivo. The target genes of the miRTarbase database which have been considered are experimentally validated by reporter assay, western blot, microarray or next-generation sequencing experiments.

The resulting ENTREZ gene IDs were used by utilizing DAVID tools (The Database for Annotation, Visualization and Integrated Discovery, v6.8, Oct 2016) [26] for a pathway enrichment analysis. The resulting genes were mapped by the DAVID tool onto known pathway resources, in our case BIOCARTA. The significant enriched pathways were qualified and ranked by false discovery rate corrected p values (alpha error: p<0.05). Background for the analysis was the entire Homo sapiens transcriptome. The enriched pathways were discussed in the context of established knowledge.

### Validation of selected microRNAs

The selected microRNAs were validated in the same cohort of patients as described for the screening procedure using quantitative real time PCR. The number of selected patients was: 14 patients with mild, 16 with severe symptoms and 16 control samples for miR-143-3p. Similar numbers were chosen for miR-148a-3p (see S3 Fig). The validation was not performed on a pool of samples instead on each individual patient sample. The protocol of cDNA synthesis and real time PCR was the same as previously explained. Delta-Ct value was calculated using the mean of miR-103a-3p, miR423-5p and miR-191-5p as reference genes. Student's t-test was used here to evaluate statistical significance with an alpha value of smaller than 0.05. The comparison was made between control group and mild and severe groups separately. Receiver operating characteristic (ROC) curve analysis were used to determine the specificity and sensitivity in distinguishing the whole group of SMV affected patients from the normal group. All these analyses and the graphs were created using GraphPad Prism 6.

### Cell cycle analysis

To investigate the impact of microRNA overexpression on the cell cycle, a DNA segment containing miR-143-3p precursor was cloned in PEGFP-C1 vector and 1 microgram of the vector was transfected to HEK-293T using lipofectamin 2000 (Invitrogen, USA) in a 24-well plates (7 × 10 4 cells per well). The transfection rate (~70%) was assessed after 24 hours by measuring the GFP signal using a fluorescent microscope. Transfected wells were subjected to RNA extraction following cDNA synthesis to evaluate if the expression of miR-143-3p was increased compared to mock vector. U6 was used as internal control and each reaction was done in duplicate.

In parallel, a flow cytometry analysis of the cell cycle with propidium iodide (PI) was performed on the transfected cells after 15-minutes of ethanol fixation. Following fixation, the cells were rinsed with PBS and stained with PI in a solution containing Triton X-100 and RNase A. The cell cycle analysis was performed using a cell sorter (BD FACSCanto II, Becton Dickinson). The flow cytometry data analysis software (Flowing software version 2.5.1), was used to determine and plot the cell cycle profile and percentage values.

## Results

### Data properties and experimental design

The differentially expressed microRNAs in serum samples of SMVs were determined using miRNome panels (Exiqon). Following RNA extraction and cDNA synthesis, quantitative PCR array of microRNAs was performed on serum samples. Four patient`s categories including normal, mild, moderate, and severe were included in the study, while the focus for generating biological insight was mainly put on a differential analysis of normal (grey) versus mild (blue) respectively severe (red, Fig 1). All groups were based on two biological replicates, each based on two independent pools of patients. The samples were analyzed on two qPCR arrays I and II with 372 and 367 microRNAs respectively. 528 microRNAs out of 739 were at least partly expressed. The majority of expressed microRNAs with a relevant Ct value were located on panel I. To protect the downstream analysis from this biased behavior, panel II was dropped. The remaining microRNAs on panel I were further filtered to have Ct values smaller than 40 in all measurements. This might also effect on/off situations by microRNA expression but the raw data inspection showed that without this correction data reproducibility drops remarkably. The resulting number of 173 microRNAs was used for further analysis.

The qPCR panel additionally included controls, inter plate calibrators (IPCs) and six different reference genes where 3 were used in the differential analysis. The delta Ct calculation are normally based on references of small RNA species from non-microRNA origin (including SNORD38B, SNORD49A, and U6) [27, 28]. These are either absent or low expressed in our samples. In a previous study of us [21], we determined the stability of an alternate group of candidate reference genes in SMVs and found miR-103a-3p, miR-423-5p and miR-191-5p to be the most stable reference genes in SMV samples. The average of the mentioned validated reference genes was used to scale the delta Ct values.

**Fig 3:**
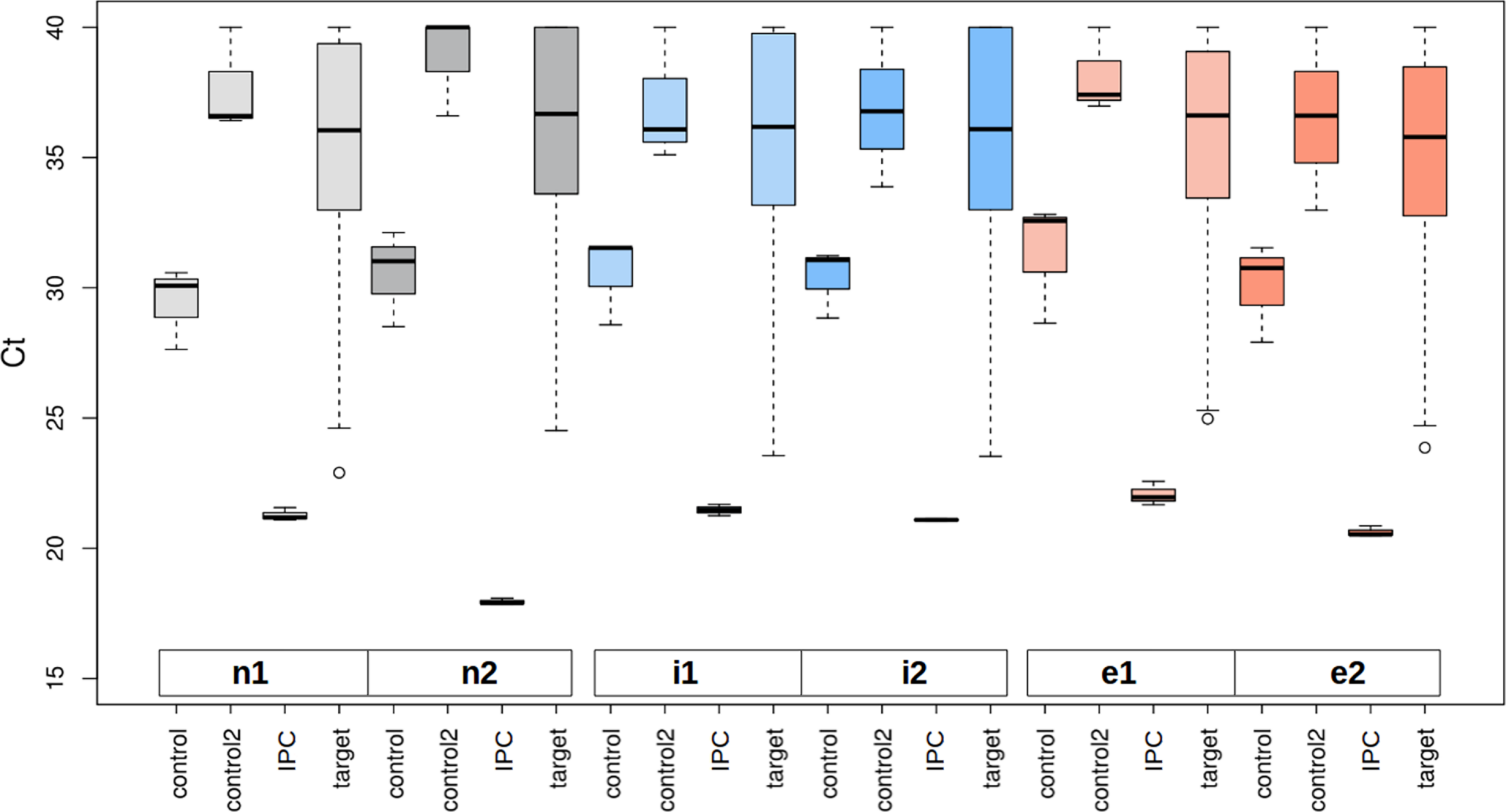
Box plots of raw data microRNA expression values and controls. On the x axis the different measurement groups for each experiment are shown: 'control' stands for reference genes including miR-103a-3p, miR-423-5p and miR-191-5p. 'control2' denotes non-miRNA coding reference genes. 'IPC' lists the inter plate calibrators. 'targets' comprises all the measured individual microRNAs. As can be seen in this figure the distribution of the target genes is nearly consistent in all tested samples even on the raw data level. The y axis denotes the Ct values.

**Fig 4:**
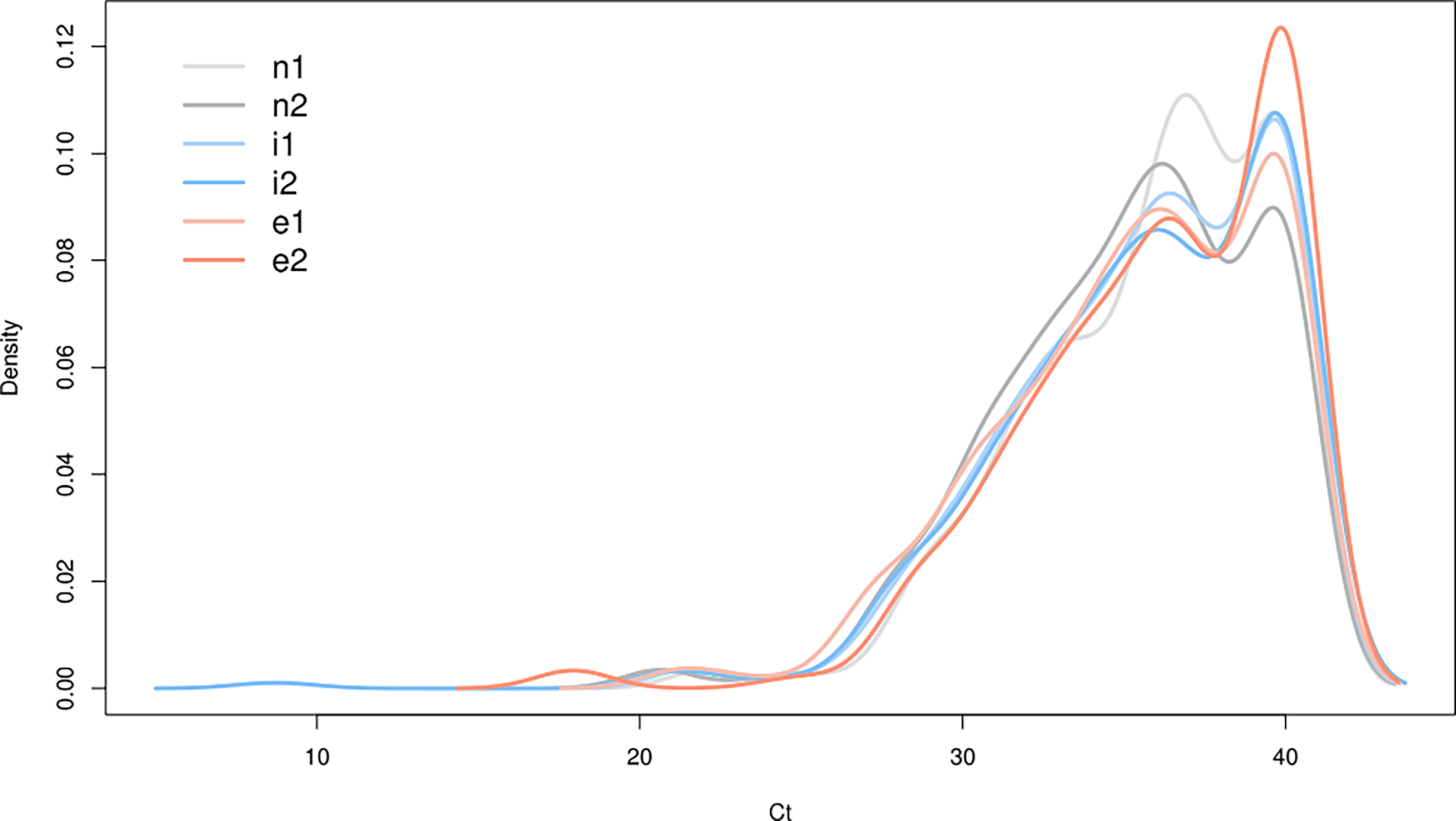
Overview on the density distributions. The target microRNAs in the different patient`s groups show a very uniform distribution on the raw data level. The y axis denotes the normalized density, while the x axis shows the Ct values. Because of the smoothing effect of the density curves Ct values above 40 show up, which is artificial (maximum is 40).

Box plots of the raw data of all sample types and replicates, split by controls and targets, are shown in Fig 3. Controls, microRNA-origin reference genes, showed moderate variations. The distribution of the target microRNAs is similar to each other which can also be seen in Fig 4. Further inspection efforts on the data behavior in different normalization situations (see S1 and S2 Figs) paved the way to the final analysis concept (Fig 2A).

### Differentially expressed microRNAs

The guiding idea for two concurrently approaches was to give more stability to the final results. The first approach was performing on the IPC adjusted and delta Ct scaled data (Fig 2A, left part of graph) while the other one works directly on the raw data (right part of the graph) which we gave preference finally due to the quality control procedures (see previous section and methods). All the analyses were performed on the normal-mild (blue) and normal-severe (red) group separately (Fig 2B).

A list of differentially expressed microRNAs was generated from each approach and each comparison (Fig 2B). The candidates were taken from the reminders in the resampling procedure (applied 10,000 times) based on significance of the resampling p-value. The mild group, 13 deregulated microRNAs were obtained from IPC/delta Ct adjusted data and 15 from raw data. The severe group comprises 35 microRNAs which showed significant changes in IPC/delta Ct and 29 in raw approach. To see if these two differential microRNA sets from IPC/delta Ct and raw approach confirm each other, we intersected the results (Fig 2B—middle part). 23 common microRNAs were obtained from severe group intersection and 12 from mild intersection which shows a good consistency of the approaches. The top candidate microRNAs are shown in Tables 1 and 2 for mild and severe group respectively (right hand approach). microRNAs are ranked based on their p-values from the most to the least significant p-values. An overview on all result sets is given in the S1 Table.

**Table 1:** Differential microRNAs (15) of the normal-mild group. The values shown here are from the right part of the workflow in Fig 2. dCt: delta Ct, FC: fold change and sampling p: sampling p value which was finally considered.

	MicroRNA Name	dCt	FC	p value	sampling p
1	hsa-miR-589-3p	5.236	0.027	0.0021	0.0006
2	hsa-miR-542-5p	4.701	0.038	0.0022	0.0014
3	hsa-miR-886-5p	4.305	0.051	0.00446	0.0020
4	hsa-miR-550-5p	3.896	0.067	0.0055	0.0030
5	hsa-miR-143-3p	-4.725	26.454	0.0070	0.0033
6	hsa-miR-520d-5p	3.311	0.101	0.0105	0.0051
7	hsa-miR-329-3p	3.416	0.094	0.0121	0.0057
8	hsa-miR-654-5p	3.101	0.117	0.0136	0.0088
9	hsa-miR-509-3p	3.310	0.101	0.0152	0.0104
10	hsa-miR-185-3p	3.425	0.093	0.0182	0.0130
11	hsa-miR-510-5p	2.961	0.128	0.0180	0.0137
12	hsa-miR-301b-3p	2.770	0.147	0.0250	0.0188
13	hsa-miR-99a-5p	-2.949	7.722	0.0366	0.0335
14	hsa-miR-202-3p	2.466	0.181	0.0393	0.0358
15	hsa-miR-184	2.431	0.185	0.0430	0.0384

**Table 2:** Differential microRNAs (29) of the normal-severe group. The values shown here are from the right part of the workflow in Fig 2. dCt: delta Ct, FC: fold change and sampling p: sampling p value which was finally considered.

	MicroRNA Name	dCt	FC	p value	sampling p
1	hsa-miR-589-3p	5.236	0.027	0.0034	0.0007
2	hsa-miR-185-3p	4.449	0.046	0.0021	0.0008
3	hsa-miR-526b-5p	4.165	0.056	0.0046	0.0017
4	hsa-miR-30b*-3p	4.380	0.048	0.0042	0.0017
5	hsa-miR-9-5p	3.705	0.077	0.0051	0.0018
6	hsa-miR-200a-3p	-3.585	11.999	0.0052	0.0019
7	hsa-miR-886-5p	4.305	0.051	0.0051	0.0024
8	hsa-miR-520d-5p	3.311	0.1011	0.0072	0.0031
9	hsa-miR-654-5p	3.101	0.117	0.0087	0.0038
10	hsa-miR-493-3p	3.086	0.118	0.0098	0.0043
11	hsa-miR-933	-3.849	14.414	0.0101	0.0047
12	hsa-miR-663a	-4.294	19.621	0.0088	0.0051
13	hsa-miR-873-5p	-2.886	7.392	0.0113	0.0053
14	hsa-miR-377-3p	2.875	0.136	0.0117	0.0064
15	hsa-miR-510-5p	2.961	0.128	0.0123	0.0075
16	hsa-miR-95-3p	2.7001	0.154	0.0179	0.0119
17	hsa-miR-143-3p	-4.115	17.333	0.0190	0.0124
18	hsa-miR-708-5p	3.310	0.101	0.0231	0.0136
19	hsa-miR-365a-3p	-4.525	23.020	0.0218	0.0150
20	hsa-miR-202-3p	2.465	0.181	0.0272	0.0232
21	hsa-miR-452-5p	2.680	0.156	0.0319	0.0248
22	hsa-miR-490-3p	2.880	0.136	0.0374	0.0299
23	hsa-miR-760	3.536	0.086	0.0356	0.0336
24	hsa-miR-376b-3p	2.385	0.191	0.0450	0.0367
25	hsa-miR-184	2.201	0.218	0.0448	0.0389
26	hsa-miR-27b-3p	-1.916	3.774	0.0486	0.0414
27	hsa-miR-362-5p	-2.235	4.708	0.0498	0.0438
28	hsa-miR-382-5p	1.9611	0.257	0.0489	0.0448
29	hsa-miR-502-5p	3.755	0.074	0.0496	0.0494

### Pathway analysis show involvement of apoptosis, cell cycle arrest and TGF-beta signaling pathways

The result sets of mild (15) and severe (29) and the intersection between mild and severe (9) were used to generate a mapping table of microRNAs to gene targets. For this task miRTarBase [25] was used. In the case of mild group 1746 non-redundant gene ids were obtained by this mapping, in the case of the severe group 2757 and in the mild-severe intersection group 773.

DAVID tools were employed successively to analyze the affected biological pathways. The gene ids were used to look on enriched pathways by the gene set enrichment approach (GSEA, DAVID tools [26]). In the case of the mild group, GSEA analysis resulted in a list of 37 cellular pathways (Table 3, all in S2 Table). The Ras and Rho pathways regulating the G1 to S Transition was one of the perspicuous pathways by these differentially expressed group. Additionally, inhibition of ribosomal biogenesis was observed, by the presence of the tumor suppressor Arf and skeletal muscle hypertrophy regulated via AKT/mTOR pathway.

**Table 3:** The 9 top affected cellular pathways influenced from the 15 microRNAs of the normal-mild comparison.

	BIOCARTA Term	Genes ID (ENTREZ)	Fold Enrichment	FDR p-value
1	h_raccycdPathway:Influence of Ras and Rho proteins on G1 to S Transition	595, 8517, 3265, 1869, 1019, 5058, 5925, 1026, 207, 1021, 898, 5295, 5594	3.8166215	3.49E-05
2	h_arfPathway:Tumor Suppressor Arf Inhibits Ribosomal Biogenesis	7291, 1869, 84172, 4193, 5925, 5294, 5295, 4609, 51082	3.963414634	7.69E-04
3	h_igf1mtorPathway:Skeletal muscle hypertrophy is regulated via AKT/mTOR pathway	3480, 5728, 5515, 1978, 3636, 5295, 207, 6194, 2475	3.567073171	0.001766927
4	h_cellcyclePathway:Cyclins and Cell Cycle Regulation	595, 1021, 1869, 993, 894, 891, 1019, 5925, 1026, 898	3.170731707	0.002174754
5	h_ctcfPathway:CTCF: First Multivalent Nuclear Factor	5728, 5515, 4089, 7049, 4193, 7048, 5294, 5295, 4609, 2475	3.170731707	0.002174754
6	h_erkPathway:Erk1/Erk2 Mapk Signaling pathway	2872, 3480, 3265, 2782, 5156, 9252, 5515, 1956, 2002, 4609, 5594	2.906504065	0.002408948
7	h_p53Pathway:p53 Signaling Pathway	595, 1869, 596, 4193, 1019, 5925, 1026, 898	3.730272597	0.002850649
8	h_telPathway:Telomeres, Telomerase, Cellular Aging, and Immortality	3480, 596, 5515, 1956, 3845, 5925, 207, 4609	3.52303523	0.00417549
9	h_tgfbPathway:TGF-beta signaling pathway	4088, 4089, 6498, 7048, 6885, 9372, 4092, 4087	3.337612323	0.005919135

In the severe case the GSEA analysis resulted in 48 pathways which are shown in Table 4 (all in S3 Table). Here the TNFR1 signaling pathway, the extrinsic pathway of apoptosis, the TFG-beta signaling pathway, the cell cycle G1/S check point were the most affected pathways.

**Table 4:** The 9 top affected cellular pathways influenced from the 29 microRNAs of the normal-severe comparison.

	BIOCARTA Term	Genes ID (ENTREZ)	Fold Enrichment	FDR p-value
1	h_tnfr1Pathway:TNFR1 Signaling Pathway	9530, 5599, 4214, 7124, 5058, 6885, 9731, 836, 4000, 4001, 835, 1676, 1677, 7186, 84823, 142	2.526724976	3.63E-04
2	h_tgfbPathway:TGF-beta signaling pathway	7040, 2033, 4088, 4089, 324, 6498, 7048, 6885, 9372, 4092, 999, 4087	2.992174313	4.54E-04
3	h_g1Pathway:Cell Cycle: G1/S Check Point	595, 7040, 1021, 993, 7027, 472, 4088, 2932, 1017, 4089, 1027, 1026, 898, 7157, 983	2.368804665	0.001372642
4	h_cellcyclePathway:Cyclins and Cell Cycle Regulation	595, 7027, 1017, 1027, 1026, 983, 1021, 993, 894, 5933, 891, 898, 896	2.463556851	0.002255077
5	h_il2rbPathway:IL-2 Receptor Beta Chain in T cell Activation	3265, 596, 6464, 5478, 8651, 3716, 207, 6198, 1399, 2885, 3667, 6777, 9021, 2353, 22806, 4609, 5594	2.065111759	0.003326904
6	h_mapkPathway:MAPKinase Signaling Pathway	4149, 4209, 3265, 5599, 1432, 8550, 6464, 2872, 7040, 7186, 4214, 673, 9252, 4215, 4293, 5058, 6885, 1326, 1385, 6667, 6197, 6198, 2885, 5597, 4790, 2353, 5598, 5879, 4609, 5594	1.633658389	0.004199785
7	h_nfatPathway:NFAT and Hypertrophy of the heart (Transcription in the broken heart)	811, 3265, 2147, 5599, 805, 58, 9421, 1432, 801, 4775, 2932, 207, 5532, 1906, 4620, 6198, 5573, 808, 4773, 1482, 5594	1.842403628	0.004474806
8	h_raccycdPathway:Influence of Ras and Rho proteins on G1 to S Transition	595, 3265, 7027, 1017, 5058, 1027, 1026, 207, 1021, 4790, 898, 5879, 5594	2.281071159	0.00499535
9	h_pyk2Pathway:Links between Pyk2 and Map Kinases	3265, 805, 1399, 5599, 4214, 1432, 2885, 6464, 801, 808, 5058, 5879, 5594	2.199604332	0.007120062

Comparing the mild and severe group, the involved pathways and their significance based orders are different. Nevertheless, some overlap concerning cell cycle arrest and TGF-beta signaling pathways exist.

In extension to the group specific pathway signatures, the core set of the 9 common microRNAs between the mild and severe group were analyzed. The rationale behind that was that a more common outcome might point to a gradual progression of the SMV disease while a complete different outcome might support independent subgroups. The resulting 25 core pathways are pointing to: telomeres, telomerase, cellular aging and immortality, influence of Ras and Rho proteins on G1 to S transition and skeletal muscle hypertrophy (Table 5, all in S4 Table). So the result is pointing more towards a gradual progression scheme.

**Table 5:** The 9 top affected cellular pathways influenced from the 9 microRNAs of the mild-severe intersection.

	BIOCARTA Term	Genes ID (ENTREZ)	Fold Enrichment	FDR p-value
1	h_telPathway:Telomeres, Telomerase, Cellular Aging, and Immortality	3480, 596, 5515, 3845, 207, 4609	5.310457516	0.003759507
2	h_raccycdPathway:Influence of Ras and Rho proteins on G1 to S Transition	595, 3265, 5058, 1026, 898, 207, 5594	4.130355846	0.005046085
3	h_igf1mtorPathway:Skeletal muscle hypertrophy is regulated via AKT/mTOR pathway	3480, 5728, 5515, 1978, 3636, 207	4.779411765	0.006156962
4	h_erkPathway:Erk1/Erk2 Mapk Signaling pathway	2872, 3480, 3265, 5156, 5515, 4609, 5594	3.717320261	0.008692161
5	h_tnfr1Pathway:TNFR1 Signaling Pathway	1677, 7124, 5058, 6885, 9731, 84823, 4001	3.717320261	0.008692161
6	h_mitochondriaPathway:Role of Mitochondria in Apoptotic Signaling	56616, 54205, 1677, 596, 331, 9731	4.344919786	0.00946723
7	h_caspasePathway:Caspase Cascade in Apoptosis	54205, 1677, 331, 9731, 84823, 4001	3.982843137	0.01383679
8	h_mapkPathway:MAPKinase Signaling Pathway	2872, 4209, 3265, 673, 8550, 5597, 5058, 6885, 5598, 6667, 4609, 5594	2.197430696	0.016110142
9	h_p53Pathway:p53 Signaling Pathway	595, 596, 4193, 1026, 898	4.685697809	0.017859453

### Confirming the expression pattern of miR-143-3p in each individual sample

Because of age related effects play a role in the SMV phenotype, and miR-143-3p has been previously shown to be involved in aging processes [29], and for being part of all analysis subgroups, miR-143-3p was selected for an independent validation.

**Fig 5:**
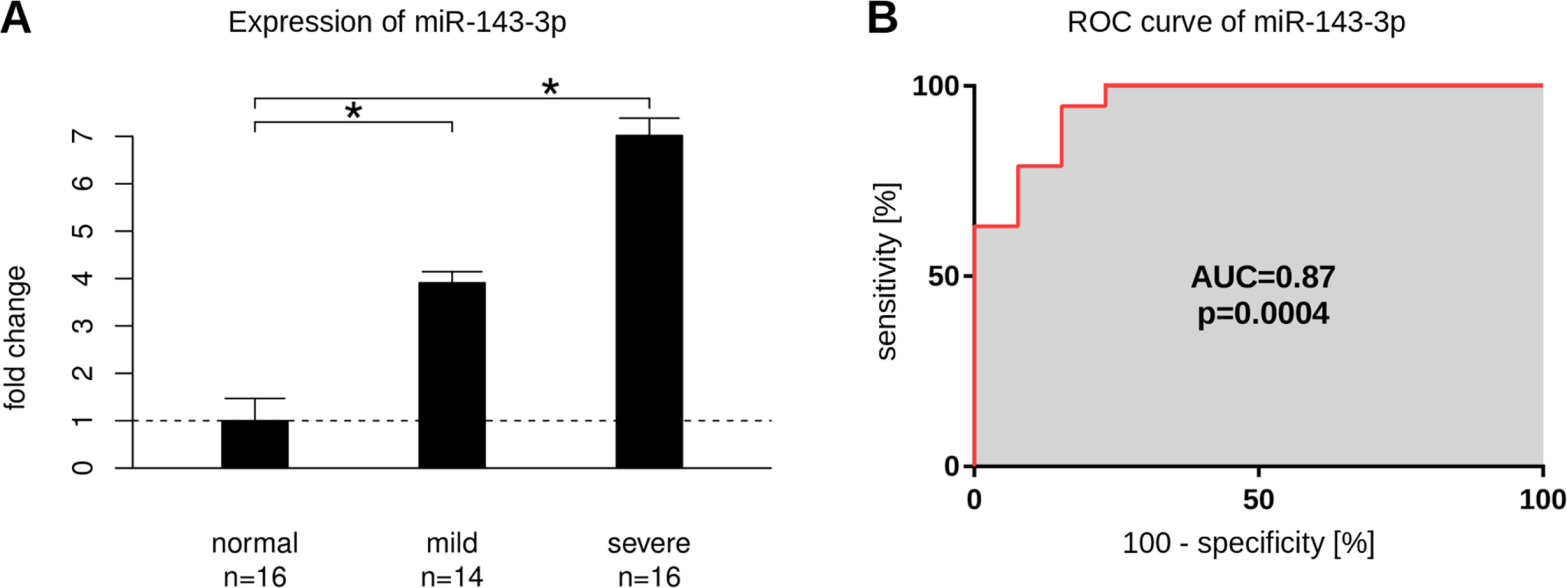
Validation of miR-143-3p expression. (A) Increased expression of miR-143-3p in either 'mild' and 'severe' group. miR-143-3p expression was up-regulated in both mild and severe groups with a fold change of 3.9 and 7.0 respectively (p = 0.005 and p = 0.1*10^−6^). The graph shows the mean values of all validated patients. The number of patients in the individual validation is slightly different from the number of patients in the pool samples. On the y axis the fold change is denoted. The star on top of the horizontal brackets indicate a significant difference. n is giving the sample number of all individually validated patients. The standard deviation is given by the top indicators. (B) The receiver-operator characteristic curve for miR-143-3p suggests this microRNA for being a suitable biomarker. miR-143-3p is able to discriminate SMV patients from control samples by an AUC of 0.87 (p = 0.0004). The x and y axis denote the percentage values of the performance parameters 'specificity' and 'sensitivity' over the full range from 0 to 100 percent.

The results showed that miR-143-3p was significantly up regulated in all individual patients both in the mild and severe group (Fold change of 3.8 and 6.6 for mild and severe respectively, Fig 5A). Additionally, the sensitivity and specificity was analyzed by the ROC method (Fig 5B). The areas under the curve (AUC) is 0.87, which denotes an acceptable performance for a potential biomarker.

As a side note, we also validated the expression level of miR-148a-3p. This microRNA has been shown to be involved in the extrinsic pathway of apoptosis and of asthma [30] which has some clinical relevance for the SMV phenotype. Despite insignificant in the presented panel analysis the expression of miR-148a-3p in the validation approach was significantly up-regulated between the normal and severe group (S3 Fig).

### Ectopic expression of miR-143-3p induces G1 cell cycle arrest

The significant rise in the expression of miR-143-3p in both, the mild and severe, groups of patients suggested a probable role in the disease pathogenesis. Therefore, the effect of miR-143-3p on cell cycle progression was investigated in HEK293T cells (usage example see [31]). HEK293T cells were transfected with the efficiency of more than 70 percent. qPCR expression analysis confirmed miR-143-3p over expression 24 h post transfection. The fold change of miR-143-3p was about 5.3 compared to mock treated cells (p = 0.008, S4 Fig). Flow cytometry analysis showed a significant accumulation of cells in the sub-G1 phase (21.6% compared to 4.1% for mock and 3.8% for untreated cells, p < 0.01, S5 Fig). This point to some role of miR-143-3p in cell cycle arrest and a potential function in cell aging events.

## Discussion

Because COPD phenotype is the most reported long-term complication of SMV patients, GOLD standard was used to categorize these patients based on their lung severity. Nevertheless, the observed COPD phenotype in the SMV patients is different from conventional COPD resulting from smoking or other etiologies. To get a better insight into the molecular situation and to identify diagnostic marker, microRNA expression was investigated to search for any differences at molecular level in these patients and also to decipher molecular mechanisms which might be responsible for long-term clinical manifestation of sulfur mustard. In this regard, the results will be discussed in separate sections: differential microRNAs, validation and cell cycle, and presumably affected pathways.

### Differential expressed microRNAs

Most of the differentially expressed microRNAs in the mild and severe category are down regulated. The minor proportion of up-regulated microRNAs comprises particularly the most significant one in both mild and severe patients: miR-589-3p (c.f. Tables 1 and 2). Recently, Wu et al showed that the expression level of miR-589-3p changes under hypoxia condition which can result in different diseases such as pulmonary artery hypertension [32]. It also has been shown that pulmonary hypertension is one of the late toxic effects of sulfur mustard exposure [33].

Another microRNA which is up regulated in our data set is miR-365a-3p. It is known that miR-365a-3p, targeting IL6/8, down-regulates the expression of IL-6 [34]. A recent publication describes an increase of the expression levels of the inflammatory markers IL-6 and -8 in the acute phase of sulfur mustard exposure, but a decrease in the chronic phase [35]. The over-expression of miR-365a-3p in our samples observed in the chronic phase, is therefore supporting the published observation. The test of this microRNA as a phase specific biomarker in future studies might be promising.

miR-143-3p, up-regulated in our samples, is involved in cell cycle regulation [36]. A study of Bonifacio et al. showed that the expression level of miR-143-3p is higher in aged fibroblast cells compared to young cells. Additionally, they showed that miR-143-3p over-expression in the fibroblasts is involved with cell cycle arrest [29]. This is consistent with the observation that the main mechanism involved in SMV pathogenesis is cell cycle regulation.

Another up regulated microRNA in our samples is miR-200a-3p. miR-200a-3p is a tumor suppressor which regulates the EMT pathway in colorectal cancer [37]. Additionally, this microRNA is involved in cell division and apoptosis processes. In 2010, Uhlmann et al. showed that miR-200a-3p is also involved in self-renewal and cell cycle regulation. They showed that miR-200a-3p is able to arrest the cell cycle at the G1 phase by down-regulating the expression of CDK6 [38]. In a further study, Li et al. showed that regulatory elements of miR-200a-3p family contain CPG island which are part of epigenetic control circuits [39]. This is of special interest, because epigenetic changes are one of the plausible mechanisms for chronic effects of SMV patients [40]. miR-200c-3p another member of miR-200 family has the same function like miR-200a-3p in EMT transition [41, 42]. But this microRNA which is also up-regulated is missing the significance threshold (p = 0.07).

miR-663a, a tumor suppressor, is another up-regulated microRNA in our study. It attenuates tumor growth and invasiveness by targeting eEF1A2 in pancreatic cancer [43]. A second role is to regulate cellular senescence under oxidative stress situation [44] or in aged cells [29,45]. To sum up, some of the up-regulated microRNAs are highlighting that cell cycle regulation might play a major role in the SMV phenotype.

### Validation and cell cycle

Molecules which own Ct value smaller than 35 denote promising biomarkers in biofluids. The up-regulated microRNA miR-143-3p reach this guiding threshold. This microRNA shows up in all nine comparisons including the mild and severe patient group. Because of this stable differential behavior and its functional background it was chosen for the validation experiment. The validation revealed that in all patients this microRNA is up-regulated and that the mild and severe group own a different expression level.

The over expression of miR-143-3p in HEK293T cells and the subsequent analysis of the cell cycle exhibit that miR-143-3p is able to arrest and accumulate cells at the sub-G1 stage. Similar observations are reported by two studies of Bonifacio and Soriano-Arroquia who showed that miR-143-3p is up-regulated in aged fibroblast [29] and myoblast cells [46]. Altogether, these findings link the miR-143-3p function additionally with cellular aging, which again is one of the observed clinical manifestations of SMV patients.

miR-148a-3p is up-regulated in individual validations only in the severe group. We selected this microRNA for a validation study because it controls the extrinsic pathway of apoptosis through the TRAIL pathway [47], which is also involved in lung diseases [48], the aging process [49] and asthma [30]. The extrinsic pathway of apoptosis can also be activated through the TNFR pathway. This functional background corresponds with the result that this pathway is only enriched in the DAVID analysis of the severe group, which owns lung complications and advanced tissue aging. Therefore this microRNA might be interesting as a discriminator in patient categorization. Nevertheless, the obvious variance between different experimental approaches points towards a certain variability in the microRNA expression analysis. So this first screening approach might need further refinement for less prominent expressed microRNAs.

### Deregulated pathways concerning mild and severe group

The established differential microRNAs in their three groups of mild, severe, and the intersection of mild-severe, are pointing to slightly different pathway systems. Similarities could be observed concerning cell cycle or TGF-beta signaling pathway.

In the mild versus normal comparison (Table 3), the most significant pathway refers to the G1 to S transition and the Ras and Rho proteins. The tumor suppressor Arf inhibits the ribosomal biogenesis and was the second ranked pathway in this category. Association of this pathway with cell cycle arrest has been previously reported [50]. Skeletal muscle hypertrophy ranked third. In SMV patients we did not report any indication for skeletal muscle hypertrophy, instead some studies refer to smooth muscle hypertrophy in lung disease of COPD patients which is responsible for air wall thickening and exacerbation of their lung function in a progressive manner [51]. Such a pathogenesis was also observed in our patients. In this context it is of interest that Shoharti et al. stated that the medication of N-acetyl cysteine can reduce bronchial muscle hypertrophy by preventing the release of inflammatory factors [52].

In the severe versus normal comparison (Table 4) the TNFR1 signaling pathway is prominently ranked. In response to external stimuli TNFR1 is activated by TNF alpha the extrinsic mechanism of apoptosis [53]. Another way of activating the extrinsic pathway is through binding FASL to FAS / DR4 [54]. In SMV patients an increased serum level of FASL can be observed [54]. This points to some importance of the apoptosis pathways for the SMV phenotype. In vitro and in vivo studies also stated that apoptosis is one of the mechanisms responsible for the SMV phenotype in a dose and time dependent manner via intrinsic and extrinsic pathways [4, 54]. Based on the presented pathway analysis as well as the findings of Pirzad [54] it might be speculated that the extrinsic pathway of apoptosis is a susceptible therapeutic target to mitigate the effects of the SMV disease.

TGF-beta signaling pathway is the second most prevalent deregulated pathway. This result is confirming previous studies [55] which investigated the expression level of TGF-beta signaling pathways in skin biopsies of SMV patients. In the study by Khaheshi et al. [55] on skin biopsies of SMVs it was shown that the expression level of different isoforms of TGF-beta and their receptors were down-regulated. In Valizadeh et al. [56] the down-regulation of TGF-beta 1- and 2-receptor in skin biopsies of SMVs points to the same pathway system.

The G1/S check point is the third most deregulated pathways in severe patients. G1 to S arrest happens if the cells encounter a lack of sufficient nutrition, the presence of stressors or DNA damage. In the latter case, it seems that damages occurred at DNA level cannot be repaired and become persistent, which in turn might lead to cell cycle arrest. Behravan et al. [57] investigated the presence of DNA breaks in DNA samples of lymphocytes of SMV patients. They showed that 25 years after sulfur mustard exposure, DNA breaks in the exposed group are significantly higher than in the control group.

### Deregulated pathways of the common results of mild and severe

Considering the lung complication to be a progressive effect of the SMV disease, it was informative to see if any common pathway was existing in these patients in mild and severe category. Hence, we searched for the common denominator between the mild and severe group. The results set between the mild and severe group of nine overlapping microRNAs points to pathways being part of cell cycle arrest, apoptosis and again aging. So this intersection supports the already established pathways from the mild and severe group.

## Conclusion

**Fig 6:**
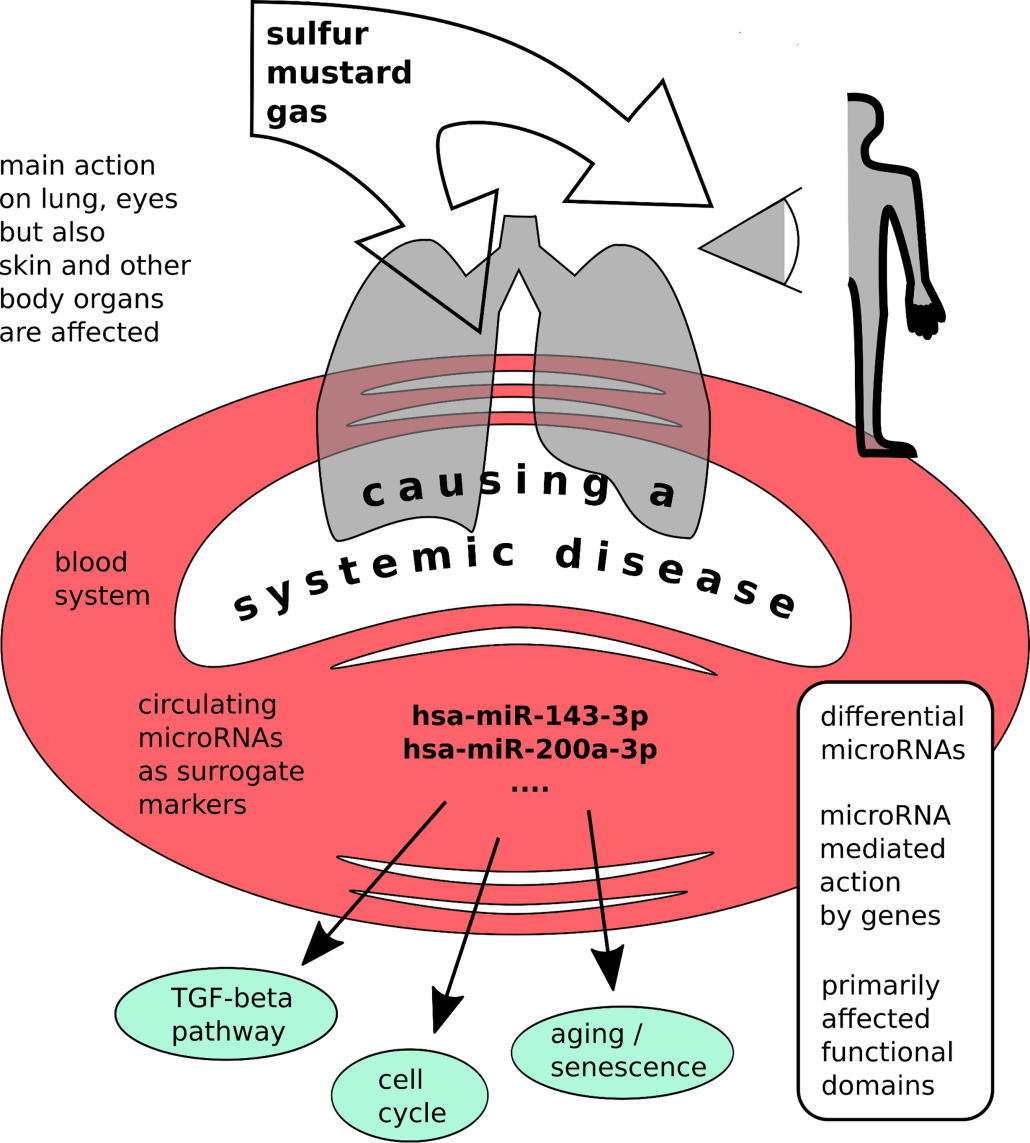
Graphical abstract.

It seems that cell cycle arrest, TGF-beta signaling pathway, apoptosis and senescence are the main affected pathways in SMV patients (Fig 6). These observations complement and extend a priory knowledge. MiR-143-3p might be eligible to serve as a diagnostic marker for SMV and be able to discriminate SMV patients from similar phenotypes. This is of importance because some of the mild patients show normal PFT values and might be misclassified.

## Supporting information

S1 Fig Conditions and data distributions. (PDF)

S2 Fig Comparative scatter plots of conditions. (PDF)

S3 Fig miR-148-3p expression on individual samples. (PDF)

S4 Fig miR-143-3p over expression. (PDF)

S5 Fig miR-143-3p - G1 cell cycle arrest. (PDF)

S1 Table All candidate sets of Fig 4B in one table. (XLSX)

S2 Table The affected cellular pathways influenced by 15 altered microRNAs in serum samples of SMVs, normal to mild comparison, BIOCARTA analysis. (XLSX)

S3 Table The affected cellular pathways influenced by 29 altered microRNAs in serum samples of SMVs, normal to severe comparison, BIOCARTA analysis. (XLSX)

S4 Table The affected cellular pathways influenced by 9 altered microRNAs in serum samples of SMVs, mild to severe intersection, BIOCARTA analysis. (XLSX)

S1 Functions Used R functions. (TXT)
