# Supplementary material for: MicroRNA expression in serum samples of sulfur mustard veterans as a diagnostic gateway to improve care
Source: PLoS One. 2018 Mar 22;13(3):e0194530. doi: 10.1371/journal.pone.0194530 (PMC5864010; doi:10.1371/journal.pone.0194530)
Supplement: S1 Fig — (PDF) [file pone.0194530.s001.pdf]

S1 Fig. Conditions and data distributions.

The panel shows seven distribution plots for the mild versus normal situation and seven for the severe versus normal situation, each for one of the conditions: raw (filtered data without normalization), IPC normalization, quantile normalization, rank invariant scaling, rank invariant normalization, deltaCt normalization, geometric mean normalization.
'p1' indicates, that only panel 1 of the Exiqon set is presented.
Every plot shows the situation for the mild/severe group (i/e, two biological pool replicates) and the normal group (n, two biological pool replicates).
The x axis denotes relative expression strength (decreasing from left to right), while the y axis denotes the density of observations.

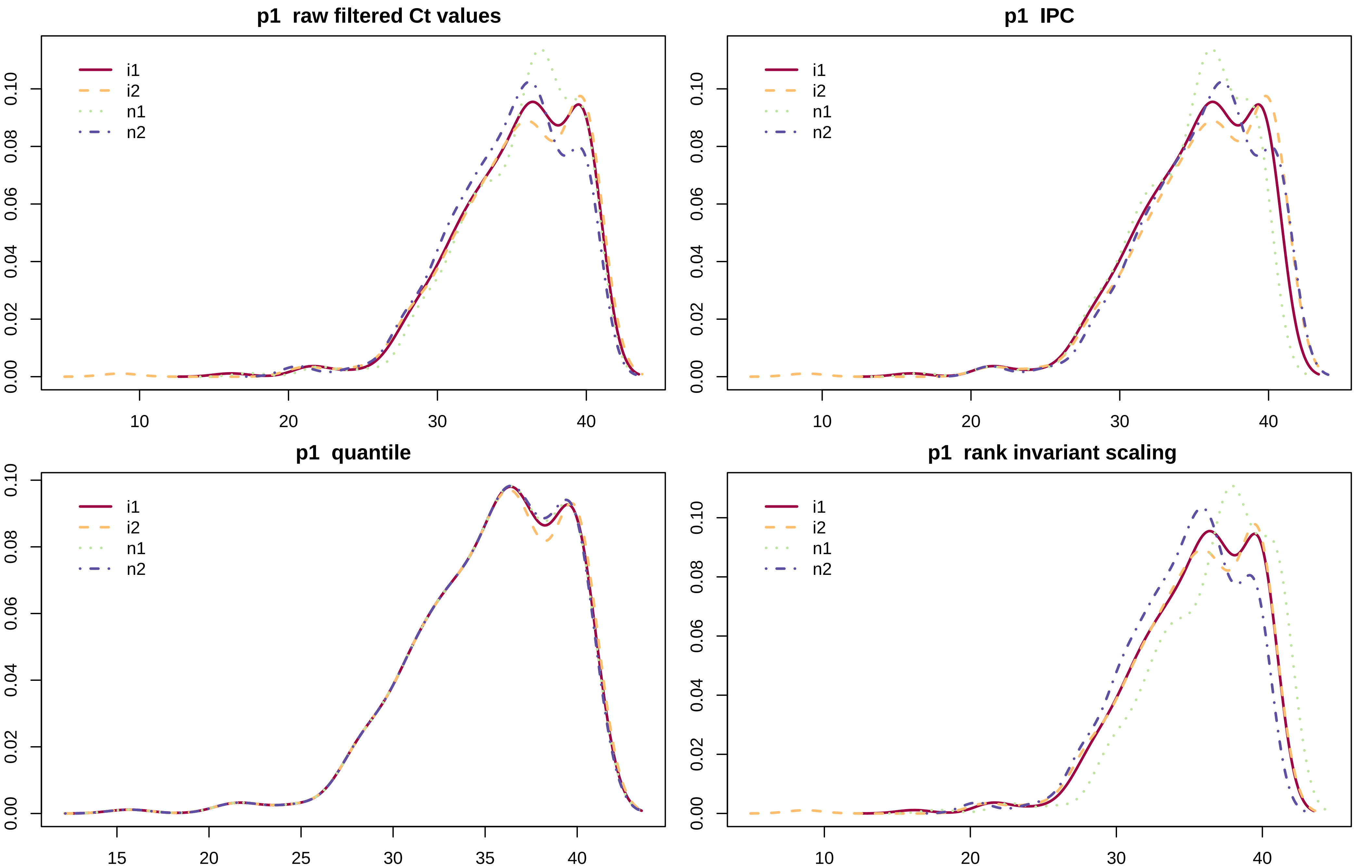

p1 raw filtered Ct values
i1
i2
n1
n2
0.00
0.02
0.04
0.06
0.08
0.10
10
20
30
40
p1 IPC
i1
i2
n1
n2
0.00
0.02
0.04
0.06
0.08
0.10
10
20
30
40
p1 quantile
i1
i2
n1
n2
0.00
0.02
0.04
0.06
0.08
0.10
15
20
25
30
35
40
p1 rank invariant scaling
i1
i2
n1
n2
0.00
0.02
0.04
0.06
0.08
0.10
10
20
30
40

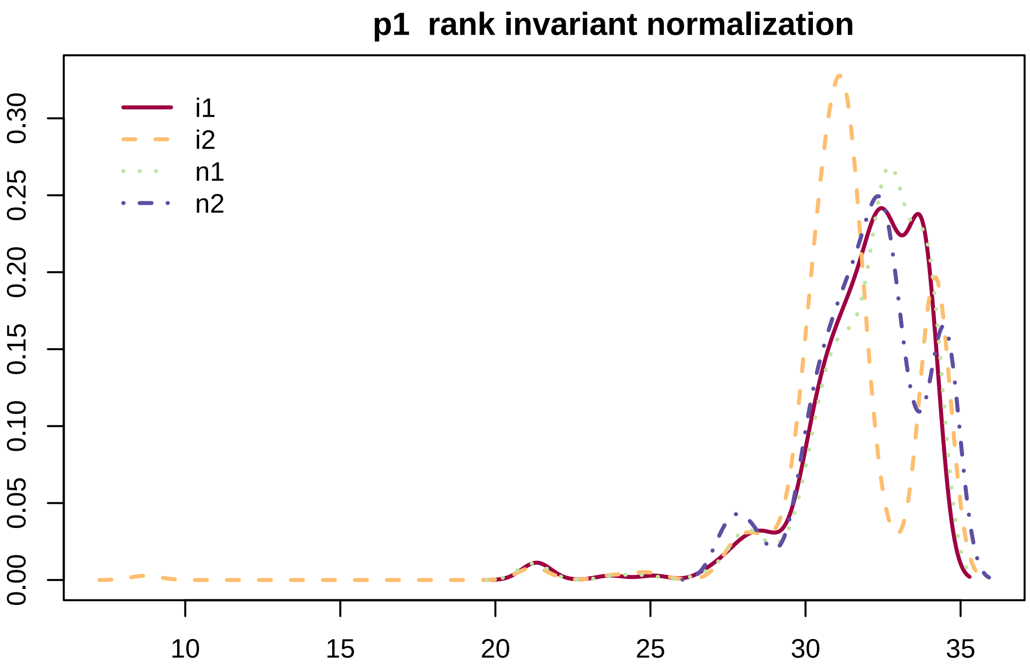

p1 rank invariant normalization
i1
i2
n1
n2
0.00
0.05
0.10
0.15
0.20
0.25
0.30
10
15
20
25
30
35

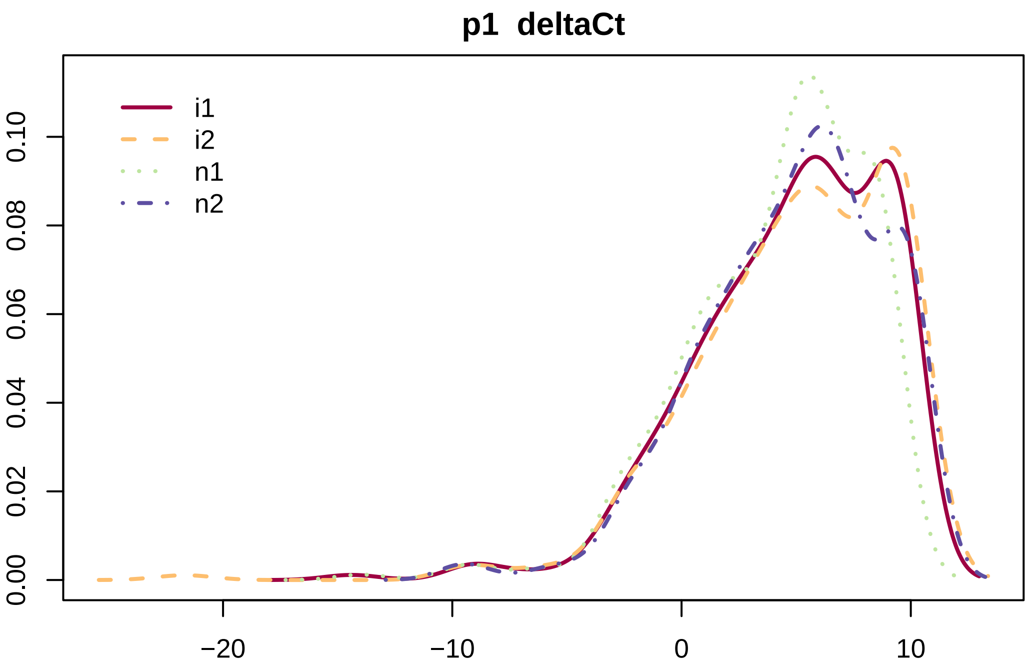

p1 deltaCt
i1
i2
n1
n2
0.00
0.02
0.04
0.06
0.08
0.10
−20
−10
0
10

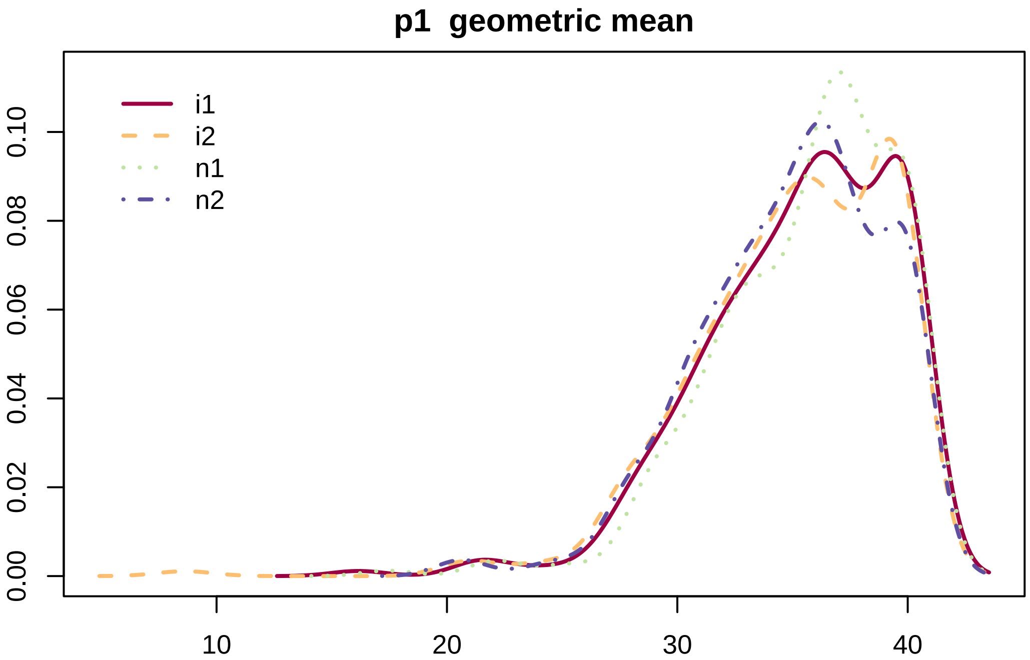

p1 geometric mean
i1
i2
n1
n2
0.00
0.02
0.04
0.06
0.08
0.10
10
20
30
40

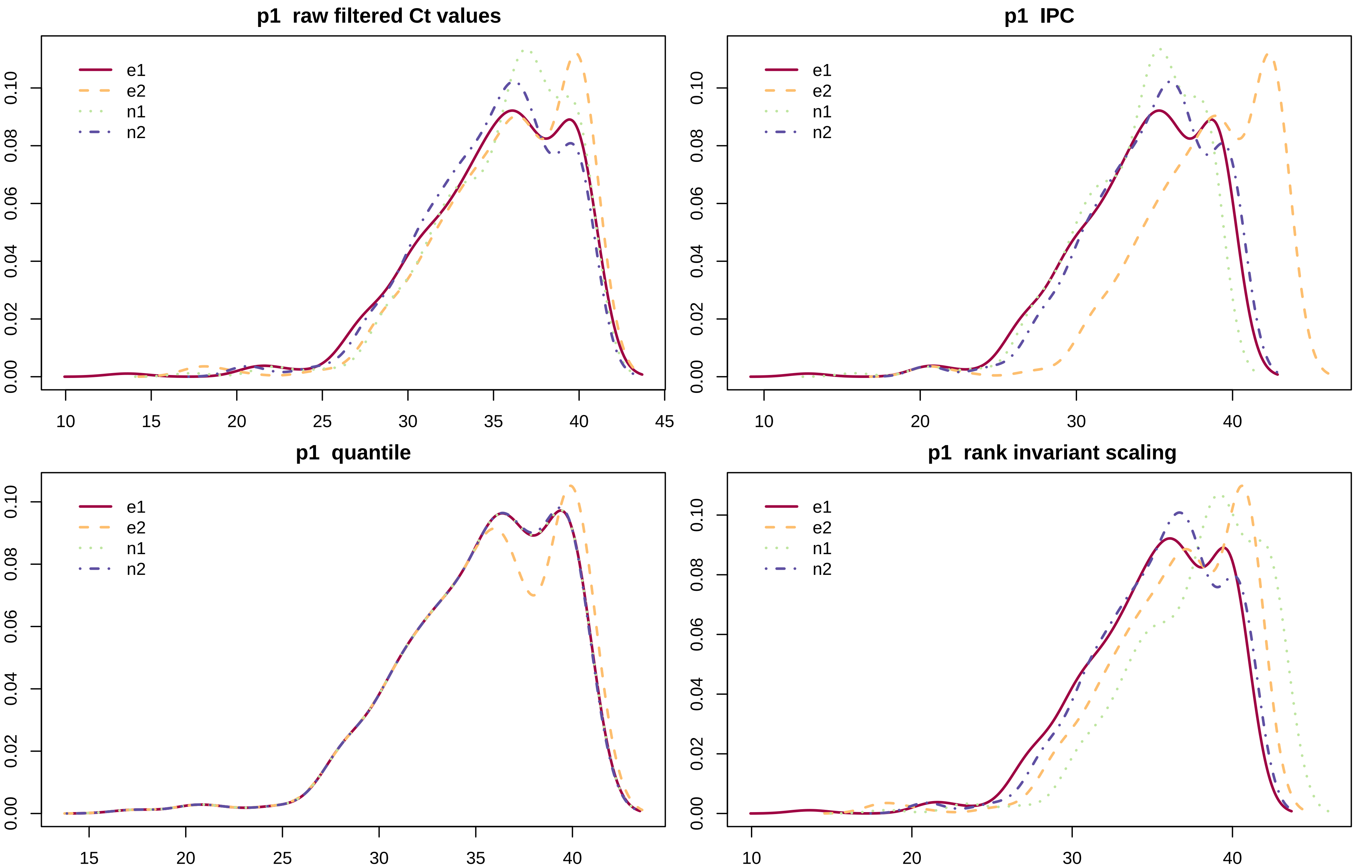

p1 raw filtered Ct values
p1 IPC
p1 quantile
p1 rank invariant scaling
e1
e2
n1
n2
0.00
0.02
0.04
0.06
0.08
0.10
10
15
20
25
30
35
40
45

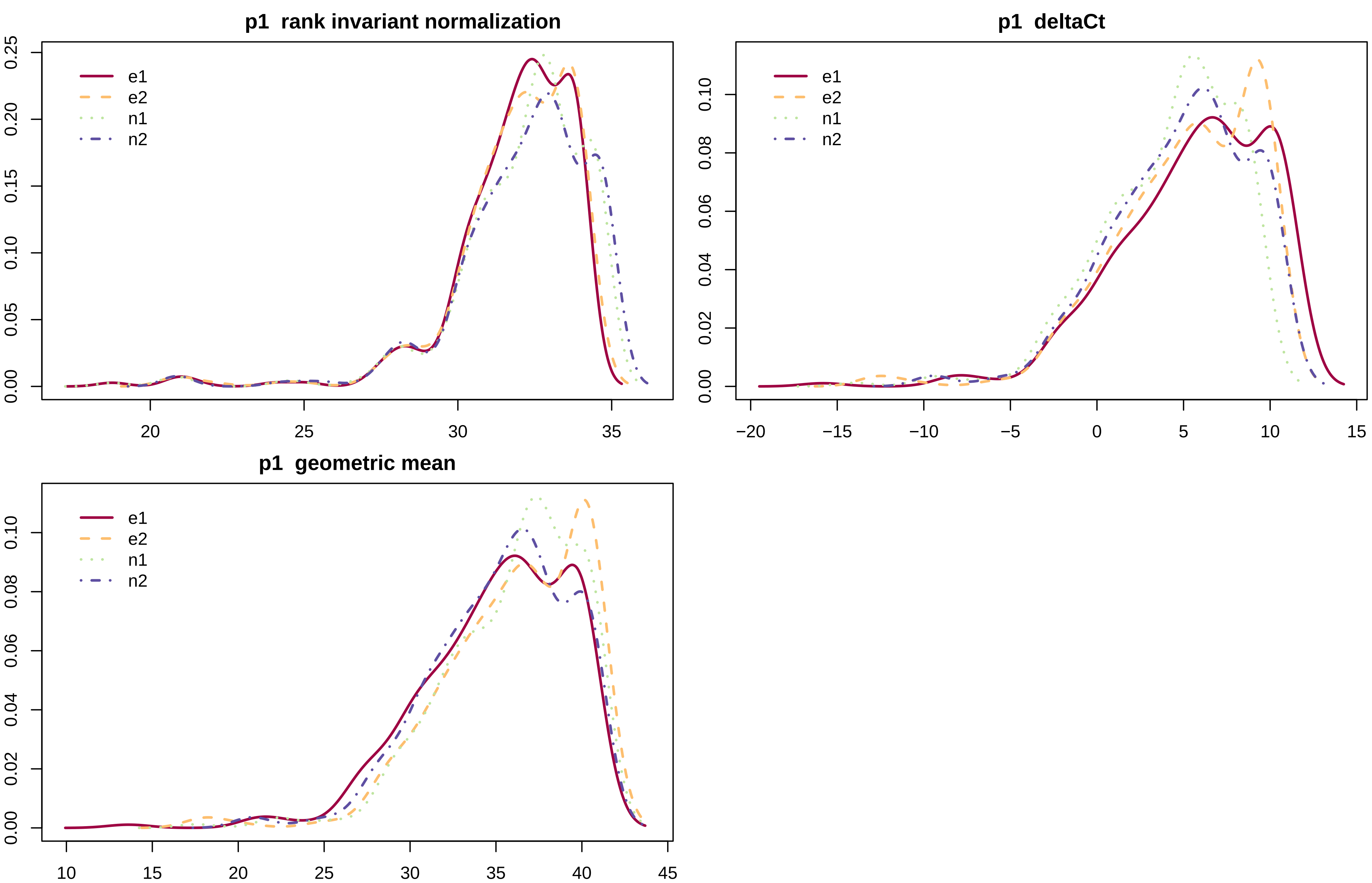
p1 rank invariant normalization
e1
e2
n1
n2
0.00
0.05
0.10
0.15
0.20
0.25
20
25
30
35
p1 deltaCt
e1
e2
n1
n2
0.00
0.02
0.04
0.06
0.08
0.10
−20
−15
−10
−5
0
5
10
15
p1 geometric mean
e1
e2
n1
n2
0.00
0.02
0.04
0.06
0.08
0.10
10
15
20
25
30
35
40
45
