# Supplementary material for: MicroRNA expression in serum samples of sulfur mustard veterans as a diagnostic gateway to improve care
Source: PLoS One. 2018 Mar 22;13(3):e0194530. doi: 10.1371/journal.pone.0194530 (PMC5864010; doi:10.1371/journal.pone.0194530)
Supplement: S2 Fig — (PDF) [file pone.0194530.s002.pdf]

S2 Fig. Comparative scatter plots of conditions.

The panel shows ten distribution plots for the mild versus normal situation and ten for the severe versus normal situation, each for one of the comparisons: raw (filtered) vs. quantile normalization, raw vs. rank invariant scaling, raw vs. rank invariant normalization, raw vs. deltaCt normalization, raw vs. geometric mean normalization, raw vs. IPC normalization and the last four panels for the sample specific perspective.
'p1' indicates, that only panel 1 of the Exiqon set is presented.
Every set of plots shows the situation either for the mild or the severe group (i/e) versus the normal group (n).
The x and y axis denotes relative expression strength (decreasing from left to right, bottom to top).

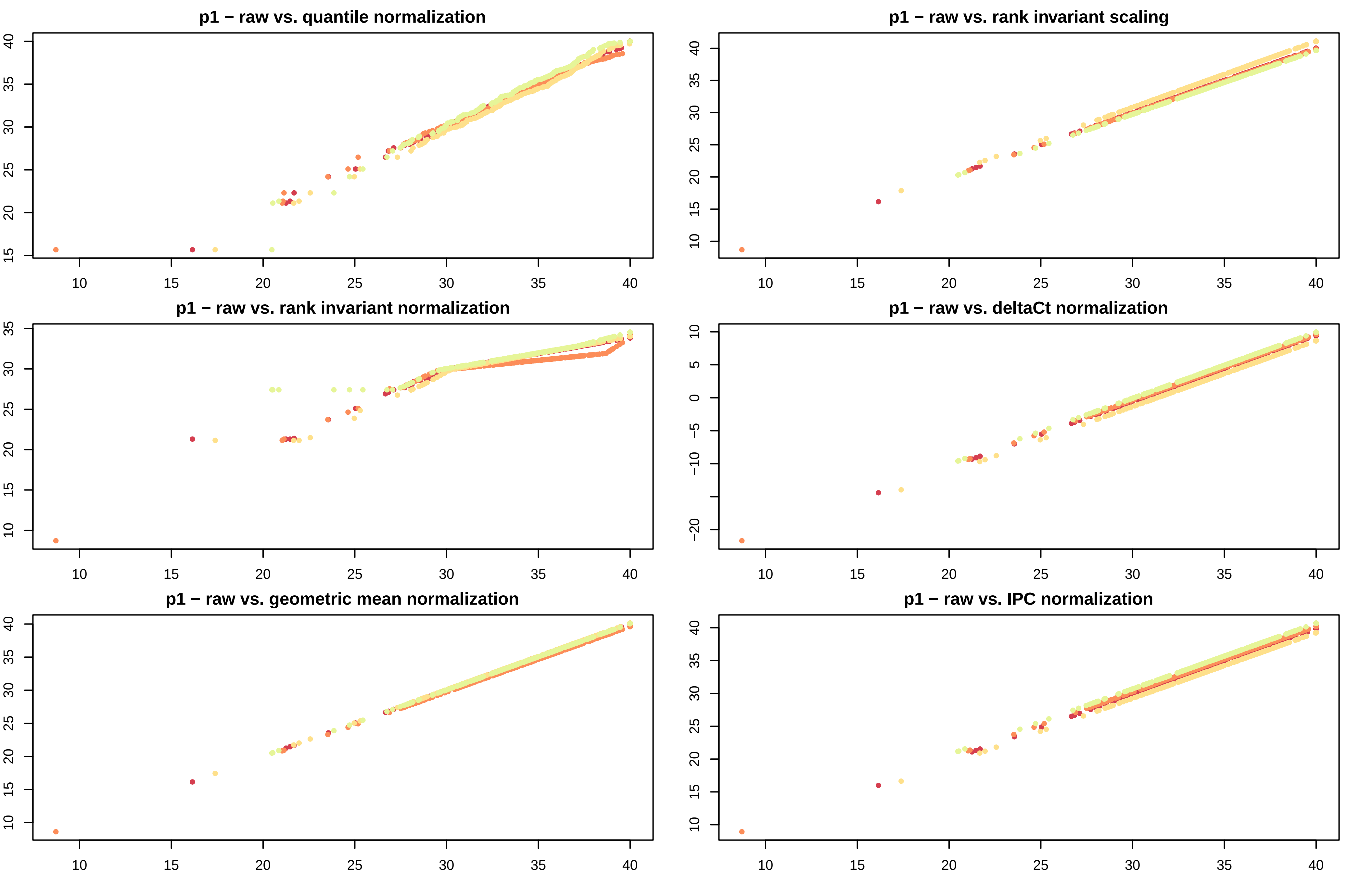
p1 − raw vs. quantile normalization
p1 − raw vs. rank invariant scaling
p1 − raw vs. rank invariant normalization
p1 − raw vs. deltaCt normalization
p1 − raw vs. geometric mean normalization
p1 − raw vs. IPC normalization
10 15 20 25 30 35 40
15 20 25 30 35 40
10 15 20 25 30 35 40
10 15 20 25 30 35
−20 −10 −5 0 5 10

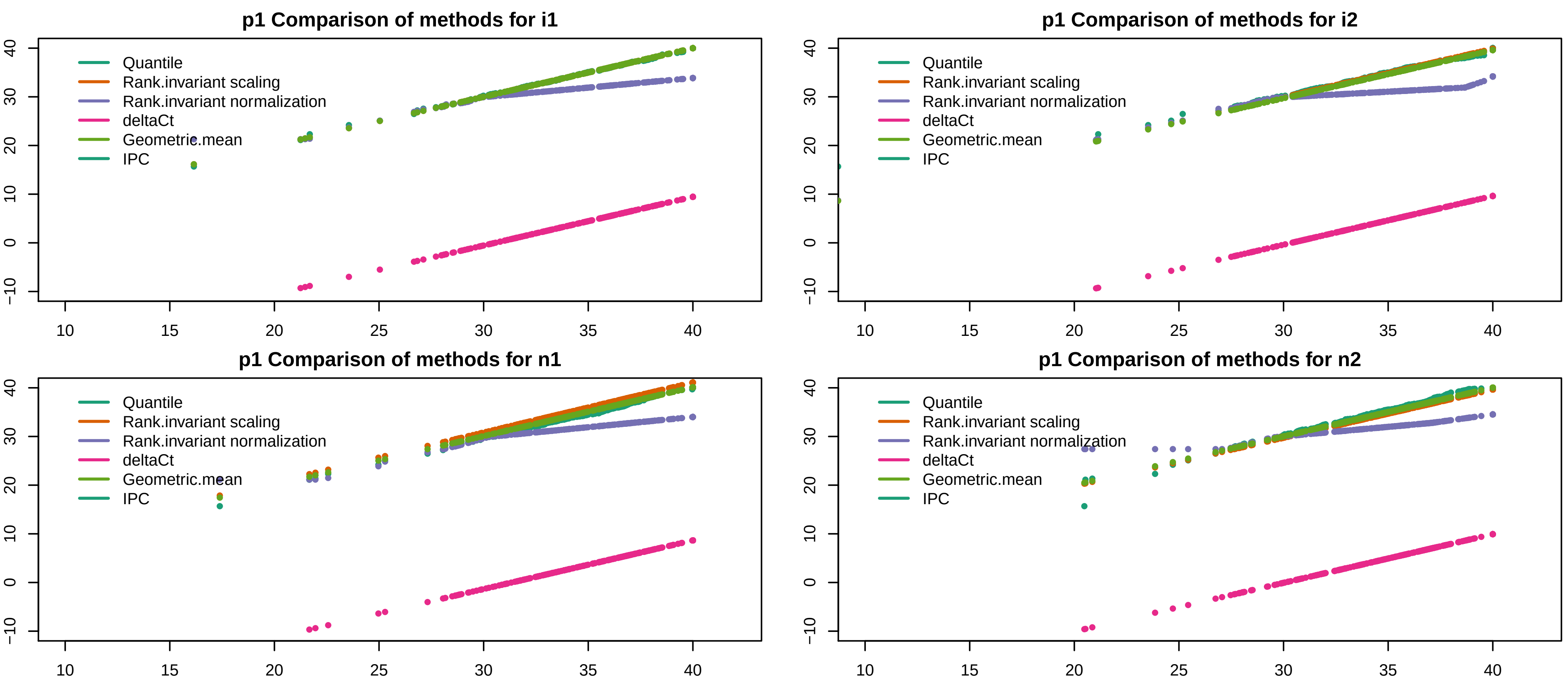
p1 Comparison of methods for i1
p1 Comparison of methods for i2
p1 Comparison of methods for n1
p1 Comparison of methods for n2
Quantile
Rank.invariant scaling
Rank.invariant normalization
deltaCt
Geometric.mean
IPC
−10
0
10
20
30
40
10
15
20
25
30
35
40

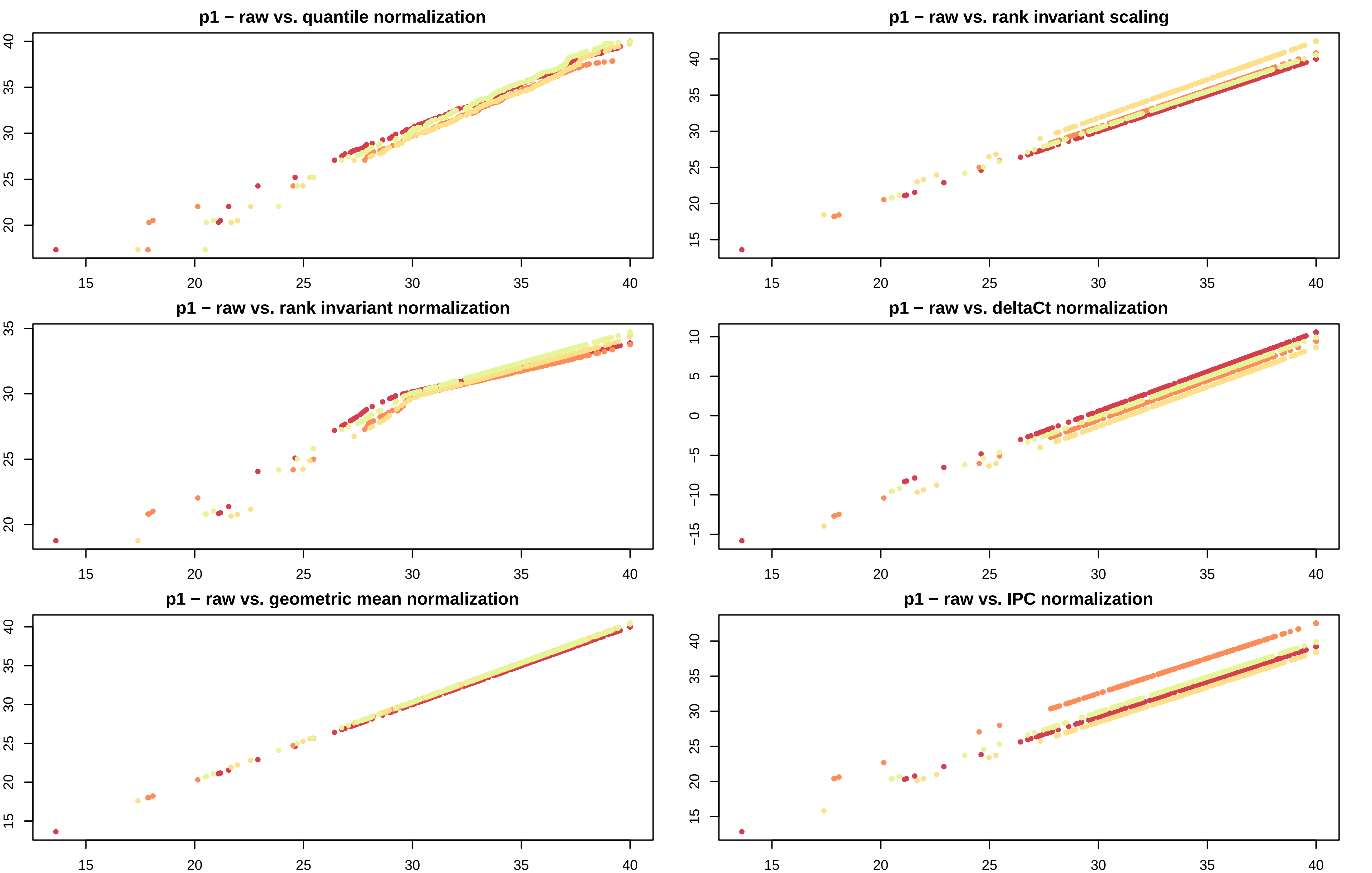
p1 − raw vs. quantile normalization
p1 − raw vs. rank invariant scaling
p1 − raw vs. rank invariant normalization
p1 − raw vs. deltaCt normalization
p1 − raw vs. geometric mean normalization
p1 − raw vs. IPC normalization

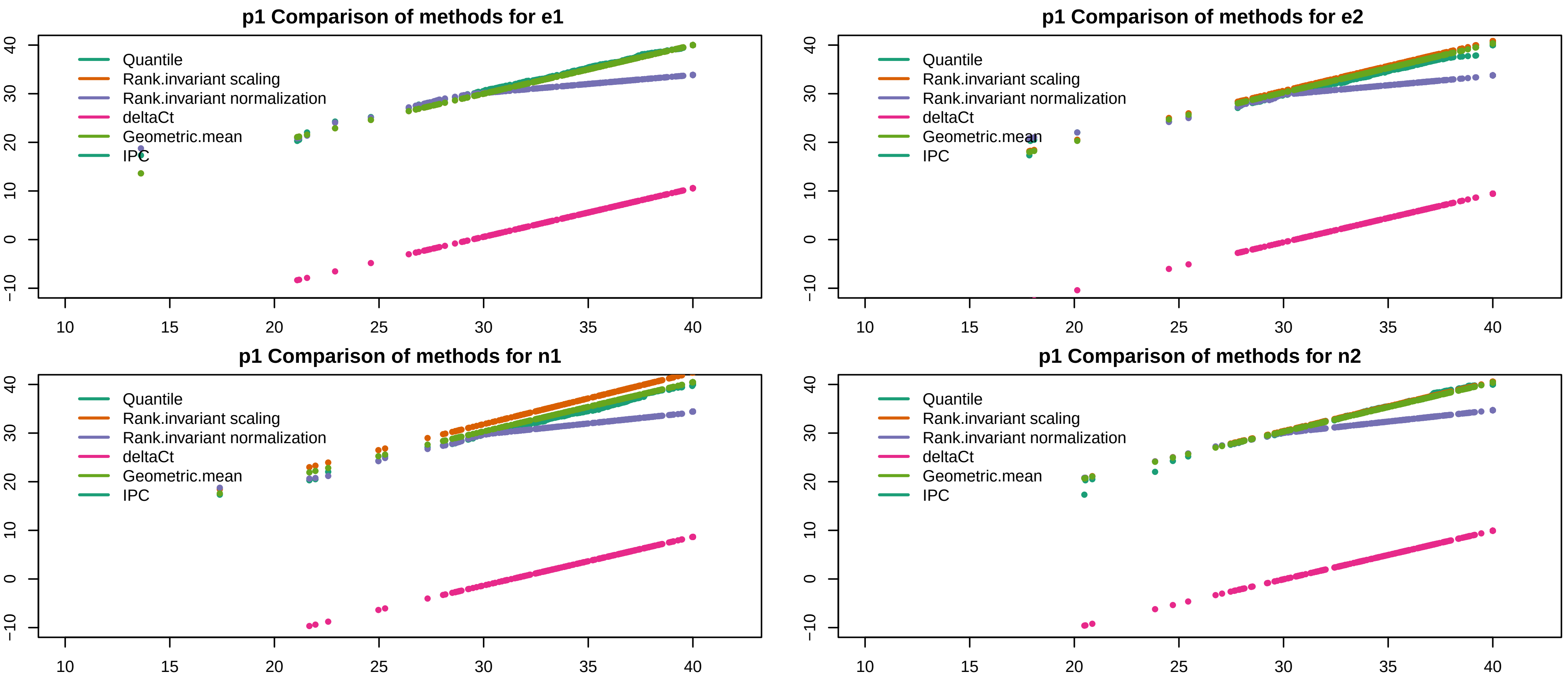
p1 Comparison of methods for e1
p1 Comparison of methods for e2
p1 Comparison of methods for n1
p1 Comparison of methods for n2
Quantile
Rank.invariant scaling
Rank.invariant normalization
deltaCt
Geometric.mean
IPC
−10
0
10
20
30
40
10
15
20
25
30
35
40
