# Supplementary material for: MicroRNA expression in serum samples of sulfur mustard veterans as a diagnostic gateway to improve care
Source: PLoS One. 2018 Mar 22;13(3):e0194530. doi: 10.1371/journal.pone.0194530 (PMC5864010; doi:10.1371/journal.pone.0194530)

S3 Fig. mir-148a-3p expression on individual samples.

(A) miR-148a-3p shows mainly in the 'severe' group an increased expression.

The fold change values of the miR-143-3p expression in both mild and severe groups were 0.6 and 2.3 respectively (p=0.231 and p=0.011). The up-regultion in normal versus severe group is moderate but significant. The graph shows the mean values of all validated patients. On the y axis the fold change is denoted. The star on top of the horizontal brackets indicate significance. n is giving the sample number of all individually validated patients. The standard deviation is given by the top indicators.

(B) The receiver-operator characteristic curve for miR-148a-3p might indicate some relevance for the 'severe' patient group.

miR-148a-3p is able to discriminate the severe group of SMV patients from control samples by an AUC of 0.77 (p=0.009). The x and y axis denote the percentage values of the performance parameters 'specificity' and 'sensitivity' over the full range from 0 to 100 percent.

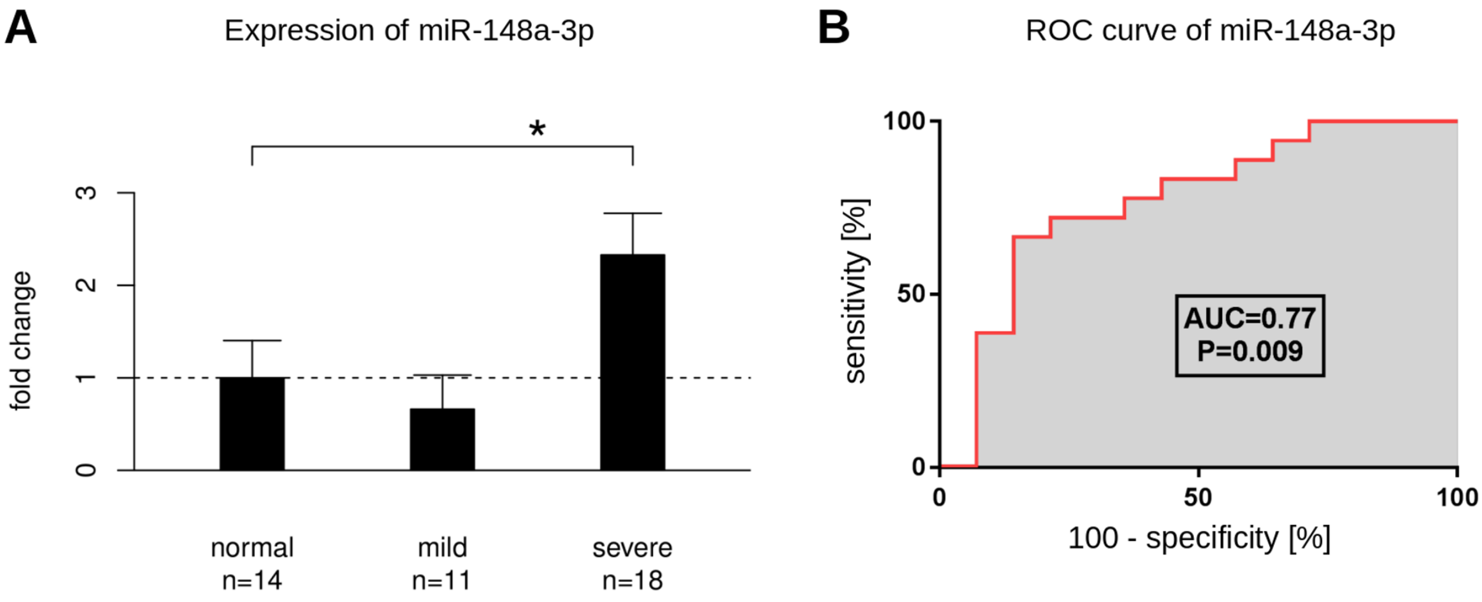

Supplement: S3 Fig — (PDF) [file pone.0194530.s003.pdf]
