# Supplementary material for: MicroRNA expression in serum samples of sulfur mustard veterans as a diagnostic gateway to improve care
Source: PLoS One. 2018 Mar 22;13(3):e0194530. doi: 10.1371/journal.pone.0194530 (PMC5864010; doi:10.1371/journal.pone.0194530)

S4 Fig. miR-143-3p over expression.

The graph shows the over expression of miR-143-3p after transfection (fold change of 5.3, p value 0.008 between mock and effect) normalized to the mean of the mock samples.
n indicates the number of samples behind the shown mean values. The standard deviation is given by the top indicators. The y axis denotes the fold change.

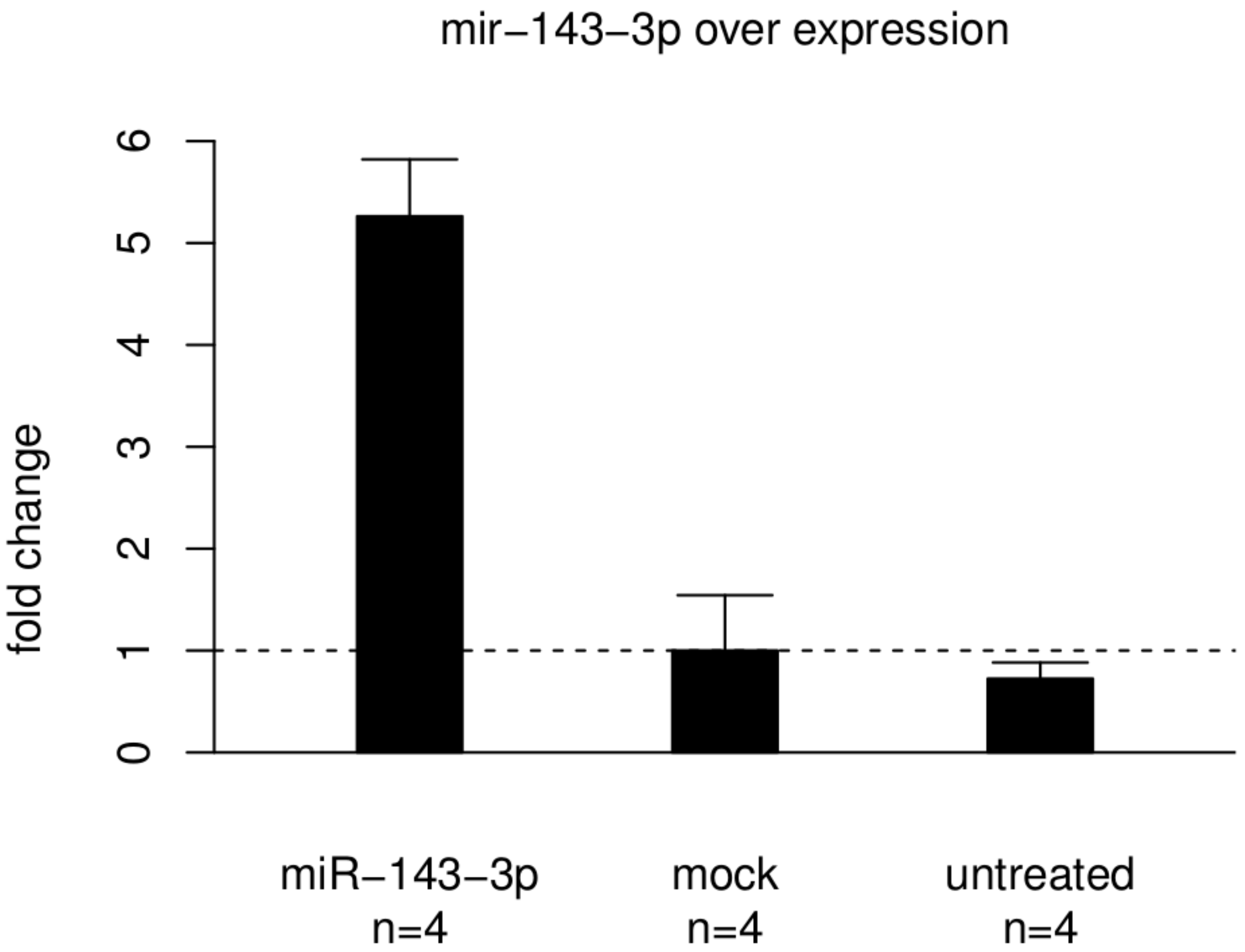

Supplement: S4 Fig — (PDF) [file pone.0194530.s004.pdf]
