# Supplementary material for: MicroRNA expression in serum samples of sulfur mustard veterans as a diagnostic gateway to improve care
Source: PLoS One. 2018 Mar 22;13(3):e0194530. doi: 10.1371/journal.pone.0194530 (PMC5864010; doi:10.1371/journal.pone.0194530)

S5 Fig. miR-143-3p - G1 cell cycle arrest.

(A) Raw data situation in the cell cycle analysis by flow cytometry. FSC (x axis) denote forward scatter while SSC (y axis) denote side scatter.
(B) Count diagram concerning certain counting windows, indicated by vertical blue lines and horizontal connector lines. The x axis corresponds to raw scatter units, respectively windows. The windows are labeled by cell cycle phases (Sub-G1, G1, S and G2). The right insert lists the percentage of counts per window. The y axis denote counts.

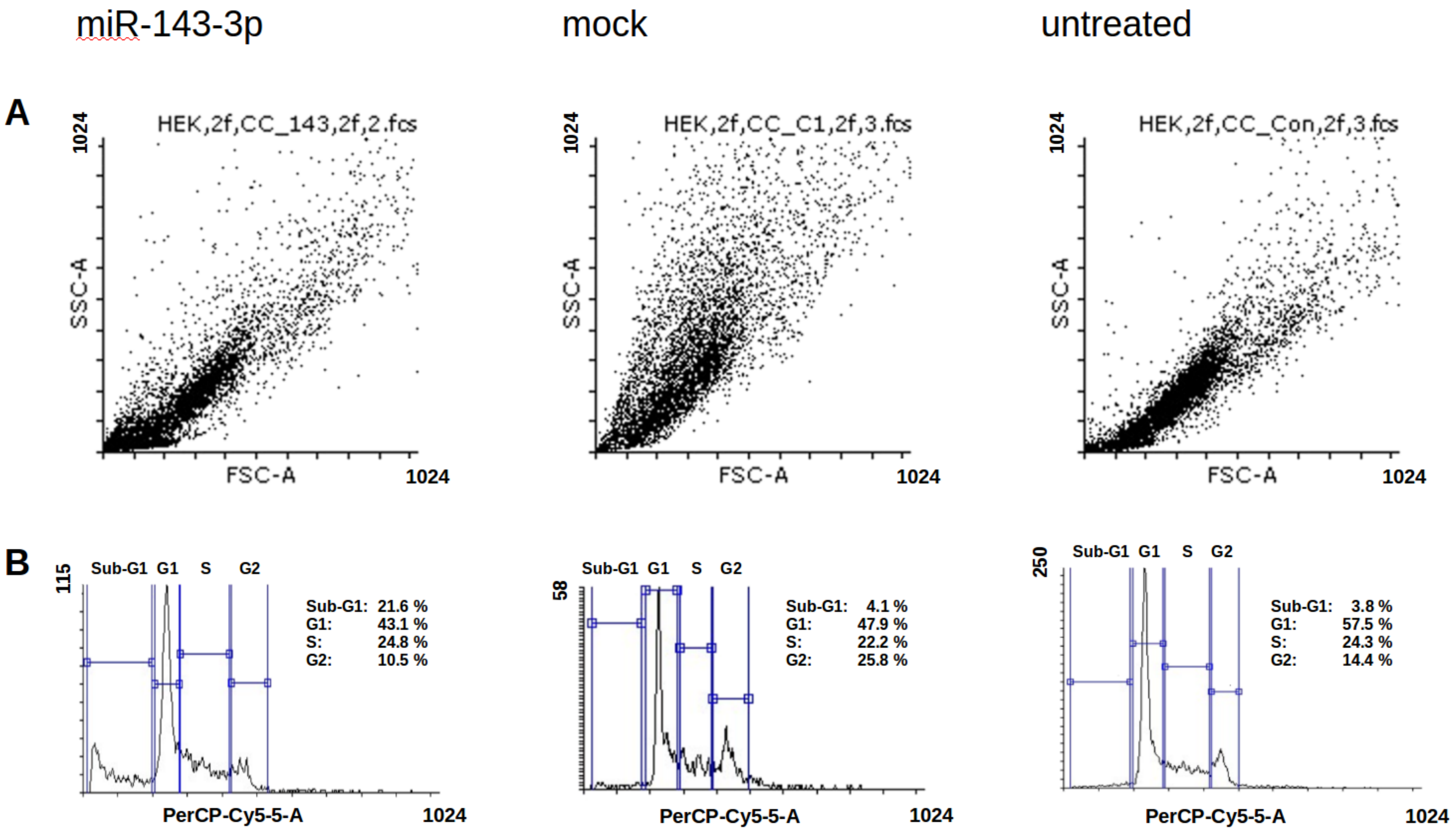

Supplement: S5 Fig — (PDF) [file pone.0194530.s005.pdf]
